# Targeting extracellular matrix remodeling sensitizes glioblastoma to ionizing radiation

**DOI:** 10.1093/noajnl/vdac147

**Published:** 2022-09-10

**Authors:** Varsha Thakur, Vijay S Thakur, Brittany Aguila, Tatiana I Slepak, Man Wang, Wei Song, Mohini Konai, Shahriar Mobashery, Mayland Chang, Ayush B Rana, Dazhi Wang, Juliano Tiburcio de Freitas, Sakir Humayun Gultekin, Scott M Welford, Michael E Ivan, Barbara Bedogni

**Affiliations:** Phillip Frost Department of Dermatology and Cutaneous Surgery, University of Miami Miller School of Medicine, Miami, Florida, USA; Sylvester Comprehensive Cancer Center, Miami, Florida, USA; Sylvester Comprehensive Cancer Center, Miami, Florida, USA; Department of Radiation Oncology, University of Miami Miller School of Medicine, Miami, Florida, USA; Sylvester Comprehensive Cancer Center, Miami, Florida, USA; Department of Radiation Oncology, University of Miami Miller School of Medicine, Miami, Florida, USA; Department of Neurological Surgery, University of Miami Miller School of Medicine, Miami, Florida, USA; Department of Chemistry and Biochemistry, University of Notre Dame, Notre Dame, Indiana, USA; Department of Chemistry and Biochemistry, University of Notre Dame, Notre Dame, Indiana, USA; Department of Chemistry and Biochemistry, University of Notre Dame, Notre Dame, Indiana, USA; Department of Chemistry and Biochemistry, University of Notre Dame, Notre Dame, Indiana, USA; Department of Chemistry and Biochemistry, University of Notre Dame, Notre Dame, Indiana, USA; Sylvester Comprehensive Cancer Center, Miami, Florida, USA; Department of Radiation Oncology, University of Miami Miller School of Medicine, Miami, Florida, USA; Sylvester Comprehensive Cancer Center, Miami, Florida, USA; Department of Radiation Oncology, University of Miami Miller School of Medicine, Miami, Florida, USA; Phillip Frost Department of Dermatology and Cutaneous Surgery, University of Miami Miller School of Medicine, Miami, Florida, USA; Sylvester Comprehensive Cancer Center, Miami, Florida, USA; Neuropathology, Division of Anatomic Pathology, University of Miami Miller School of Medicine, Miami, Florida, USA; Sylvester Comprehensive Cancer Center, Miami, Florida, USA; Department of Radiation Oncology, University of Miami Miller School of Medicine, Miami, Florida, USA; Department of Neurological Surgery, University of Miami Miller School of Medicine, Miami, Florida, USA; Phillip Frost Department of Dermatology and Cutaneous Surgery, University of Miami Miller School of Medicine, Miami, Florida, USA; Sylvester Comprehensive Cancer Center, Miami, Florida, USA

**Keywords:** GBM, MT1-MMP, (R)-ND336, radiation resistance, radio-sensitization

## Abstract

**Background:**

The median survival of Glioblastoma multiforme (GBM) patients is 14+ months due to poor responses to surgery and chemoradiation. Means to counteract radiation resistance are therefore highly desirable. We demonstrate the membrane bound matrix metalloproteinase MT1-MMP promotes resistance of GBM to radiation, and that using a selective and brain permeable MT1-MMP inhibitor, *(R)*-ND336, improved tumor control can be achieved in preclinical studies.

**Methods:**

Public microarray and RNA-sequencing data were used to determine MT1-MMP relevance in GBM patient survival. Glioma stem-like neurospheres (GSCs) were used for both in vitro and in vivo assays. An affinity resin coupled with proteomics was used to quantify active MT1-MMP in brain tissue of GBM patients. Short hairpin RNA (shRNA)-mediated knockdown of MT1-MMP and inhibition via the MT1-MMP inhibitor *(R)*-ND336, were used to assess the role of MT1-MMP in radio-resistance.

**Results:**

MT1-MMP expression inversely correlated with patient survival. Active MT1-MMP was present in brain tissue of GBM patients but not in normal brain. shRNA- or *(R)*-ND336-mediated inhibition of MT1-MMP sensitized GSCs to radiation leading to a significant increase in survival of tumor-bearing animals. MT1-MMP depletion reduced invasion via the effector protease MMP2; and increased the cytotoxic response to radiation via induction of replication fork stress and accumulation of double strand breaks (DSBs), making cells more susceptible to genotoxic insult.

**Conclusions:**

MT1-MMP is pivotal in maintaining replication fork stability. Disruption of MT1-MMP sensitizes cells to radiation and can counteract invasion. *(R)*-ND336, which efficiently penetrates the brain, is therefore a novel radio-sensitizer in GBM.

**Key Points**

- MT1-MMP mediates radio-resistance in GBM.
- MT1-MMP inhibition acts as a radio-sensitizer.
- (R)-ND336 synergizes with radiation, doubling the survival of tumor-bearing animals.
- Inhibition of MT1-MMP represents a novel approach in the treatment of GBM.

**Importance of the Study**

Surgery followed by chemoradiation is the mainstay of treatment for GBM. However, tumor progression and recurrence typically occur for the emergence of cells resistant to chemoradiation, leading to a <5% survival at 5 years. Here we highlight a novel mechanism of radio-resistance that can be exploited therapeutically. MT1-MMP is highly expressed in GBM and inversely correlates with patient survival. Active MT1-MMP was present in brain tissue of GBM patients but was not observed in control brains. Targeting MT1-MMP with the selective and brain permeable inhibitor *(R)*-ND336, doubles the survival of brain tumor-bearing animals. *(R)*-ND-336 is anticipated to enter a phase I clinical trial for the treatment of diabetic foot ulcers and is undergoing Investigational New Drug (IND)-enabling studies, which can accelerate its translation to the clinic for GBM patients. *(R)*-ND336 has potential to be impactful in conjunction with radiation or for patients that have relapsed.

Glioblastoma (GBM) is the highest grade and most lethal primary brain tumor with an average survival of 12–15 months and an overall <5% survival at 5 years^1^ despite the most aggressive treatment. Surgery, with maximal safe resection followed by radiotherapy with the concurrent use of the alkylating agent temozolomide (TMZ) is the mainstay of treatment for GBM. However, limitations of radiation therapy include permanent neuronal damage, necrosis, and resistance. Addition of TMZ to radiotherapy increases survival by 2.5 months on average, compared to radiotherapy alone.^2^ Despite these aggressive treatments, outcomes remain poor, with more than 90% recurrence.^3^ Currently, there are no approved treatments to improve the sensitization of GBM to the current therapy regime. Minimal improvement in the overall survival of GBM has been made in the last 20 years, underlying the need to find novel therapeutic targets.

The membrane bound matrix metalloproteinase MT1-MMP is involved in cancer cell invasion in several tumor types, via processing of the basement membrane and extracellular matrix (ECM).^4^ MT1-MMP also promotes tumorigenesis by activating effector MMPs (eg, MMP2, MMP13) and growth factors (eg, EGF, CD44, Notch1).^5–7^ Thus, MT1-MMP is now regarded as a critical player in tumor growth and dissemination for several cancers, including GBM.^8^ For example, inhibition of MT1-MMP in the U251 GBM line has been shown to improve survival of animals with xenograft tumors^9^; and to improve temozolomide treatment in U87 glioma derived tumors.^9^ Finally, MT1-MMP was shown to increase glioma stemness via activation of Dll4-Notch3 signaling.^10^ Overall, these studies support a role for MT1-MMP in both GBM progression and a potential role in therapy response.

How MT1-MMP may regulate tumor responses to therapy is yet to be determined. We have previously shown that, in addition to its canonical role in invasion, MT1-MMP confers radio- and chemotherapy-resistance in breast cancer via ECM remodeling; and that inhibition of MT1-MMP sensitizes breast tumors to these therapies.^11^ Here we demonstrate that inhibition of MT1-MMP in patient derived GSCs is sufficient to inhibit invasion in vitro; and, importantly, MT1-MMP inhibition sensitizes GSCs to radiation, both in vitro and in vivo. We show that brain tumors originated from GSCs in which MT1-MMP was inhibited genetically or pharmacologically, respond significantly better to radiation therapy resulting in extended survival. Therefore, MT1-MMP represents a novel therapeutic target that can simultaneously block invasion and enhance radiotherapy response of GBM patients.

## Materials and Methods

### Cell Lines and Reagents

Neurosphere glioma stem-like cell lines GBM0821 and 0913 (hereafter called 821-GSCs and 913-GSCs) were a gift of Dr. Angelo Vescovi (University of Bicocca, Milan).^12^ GBM1 and GBM12 are from Dr. Ivan’s repository and were collected under IRB #20190521 (summary of repository in Supplementary Figure 1B). d-Luciferin came from Gold Biotechnology. Stable MT1-MMP inhibition was performed with lentiviral shRNA pLKO.1 clones: TRCN0000050855 and TRCN0000050856, which we have previously characterized^13^ (Sigma). shRNA GFP was used as control. Recombinant MMP2 and MT1-MMP were purchased from R&D Systems and used at 10 ng/ml and 20 ng/ml, respectively. Quantitative real-time PCR (qRT-PCR) was performed using Power SYBR Green PCR Master Mix from Thermo Fisher Scientific and normalized to either β-actin or GAPDH. Primer sequences:

MT1-MMP F: GGCTACAGCAATATGGCTACC; MT1-MMP R: GATGGCCGCTGAGAGTGAC

MMP2 F: TGACAGCTGCACCACTGAG; MMP2 R: ATTTGTTGCCCAGGAAAGTG;

MMP9 F: GGGACGCAGACATCGTCATC; MMP9 R: TCGTCATCGTCGAAATGGGC.

### Human Tissue

Human brain cortex tissue specimens from 4 donors and 16 primary GBM samples were obtained from the NeuroBioBank Brain and Tissue Repositories of the National Institute of Health (Bethesda, MD) under a Material Transfer Agreement. Tissues originated from the Harvard Brain Tissue Resource Center, University of Maryland, Mt. Sinai Hospital, and the NIH Brain and Tissue Repository-California, Human Brain and Spinal Fluid Resource Center, VA West Los Angeles Medical Center, Los Angeles, California.

### Affinity Resin

The affinity resin was synthesized in 14 synthetic steps as described previously.^14^ Brain samples (~100 mg) were weighed and homogenized in 1 ml of cold lysis buffer (25 mM Tris-HCl pH 7.5, 100 mM NaCl, 1% v/v Nonidet P-40 and EDTA-free protease inhibitor cocktail (Pierce) using a Bullet blender (Next Advance) in the cold. Homogenates were centrifuged at 20 000×*g* for 30 min at 4°C and the supernatants were stored at −80°C. A 100-µl aliquot of the tissue extract was mixed with 100 μl of the affinity resin and 400 μl of carbonate bicarbonate buffer (50 mM Tris-HCl pH 7.5, 150 mM NaCl, 5 mM CaCl_2_ and 0.02% Brij-35) at 4°C for 2 h with rotation. After centrifugation (10 000×*g*, 12 min), the supernatant was removed, the resin beads were washed with 1 ml of carbonate bicarbonate buffer 3× and centrifuged (6000×*g*, 1–2 min). The pellet was mixed with 21 µl of 50 mM ammonium bicarbonate, 1 µl of internal standard (yeast enolase 10 nmol/ml) and 3 µl of 100 mM dithiothreitol in water. The resin-bound proteins were reduced at 65°C for 30 min. Iodoacetamide (3 µl of 100 mM in water) was added, and the alkylation was performed at room temperature for 20 min in the dark. Trypsin (2 μl of 0.1 μg/μl in 50 mM ammonium bicarbonate) was added and digestion was performed for 18 h at 37°C with shaking. Following trypsin digestion, samples were centrifuged (10 000×*g*, 2 min), and the supernatant was desalted using Millipore ZipTip^®^ C18 (EMD Millipore Corp.) and concentrated to dryness on a miVac concentrator (Genevac Ltd, Suffolk, UK). The residue was re-suspended in 20 μl of water containing 1% formic acid. A 2-µl aliquot of the samples was analyzed on a Thermo LTQ Velos Orbitrap mass spectrometer (Thermo Fisher Scientific, Waltham, MA, USA) using positive-electrospray ionization. A nano LC BEH130 C18 column (1.7 µm, 100 µm i.d. × 100 mm, Waters Corp., Milford, MA) was used. The mobile phase consisted of: 0–5 min, 99% *A*; 5–7 min, 99–90% *A*; 7–37 min, 90–60% *A*; 37–38 min, 60–15% *A*; 38–48 min, 15% *A*; 48–49 min, 15–99% *A*; 49–60 min, 99% *A*, where *A* = 0.1% formic acid and 2% acetonitrile in water, *B* = 0.1% formic acid and 2% water in acetonitrile. The flow rate was 1.2 μl/min. For identification, the Uniprot potein databsse was used. For quantification, a 2-μl aliquot of the sample was injected onto a nanoACQUITY UPLC C18 column (1.8 μm, 100 µm i.d. × 100 mm, Waters Corp., Milford, MA). The mobile phase consisted of 12-min elution at 600 μl/min with 2% acetonitrile/0.1% formic acid/water, followed by a 60 min linear gradient to 35% acetonitrile/0.1% formic acid/water. Samples were analyzed on a ABSciex QTrap 6500 mass spectrometer (ABSciex, Framingham, MA, USA) running in ion trap IDA mode coupled to a two-dimensional Eksignet Ultra NanoUPLC system, consisting of a nanoLC ultra 2D pump and a nanoLC AS-2 autosampler (Eksignet, Dublin, CA, USA). The mass spectrometer was operated in the positive-electrospray ionization mode. The following conditions were used: curtain gas 20 units, ion spray voltage 2350 V, ion source gas 110 units, ion source gas 20 units, declustering potential 100 units, entrance potential 10 units, collision cell exit potential 40 units. Three peptides (custom-synthesized by GenScript) per MMP/ADAM were used for identification and quantification (Supplementary Figure 2). Calibration curves containing known amounts of synthetic peptides (Genscript) in human plasma were processed as described for the brain samples, except using 100 µl of Sepharose resin instead of the affinity resin. Quantification of the levels of active MMPs/ADAMs was relative to internal standard. Three peptides per MMP/ADAM were used, with three transitions as qualifiers to identify the protein and three transitions as quantifiers to quantify MMPs/ADAMs.

### (R)-ND-336 Synthesis

(*R*)-ND-336 was synthesized as described previously,^15^ with a purity of 98.3%. For animal studies, (*R*)-ND336 was dissolved in distilled water/DMSO (50/50 ratio) at a concentration of 10 mg/ml and sterilized through a 0.2µ filter.

### Invasion Assays

XCELLigence.—Invasion was performed as per the manufacturer’s instructions. Briefly, 10^4^ 913-GSCs and GBM1 cells were plated on top of uncoated or 0.4% Growth Factor Reduced Matrigel/10 µg/ml hyaluronic acid (HA) coated wells (CIM plate 16). HA was used as it is a major component of brain ECM. Cells were left at room temperature for 30 min to settle and then the plates were placed into an xCELLigence RTCA DP instrument (ACEA Bioscience). Measurements were taken every 15 min for 48 h or up to 6 days. Cell Index (CI) curves normalized to initial seeding were recorded. *3D Decellularized Brain matrix:* 10^4^ 913-GSC cells were embedded in a 3D decellularized mouse brain matrix, derived as in,^16,17^ which retains the architecture and major components of brain ECM. Briefly, *Nu/nu* mouse brains were cut into small pieces and submerged in decellularizing solution [0.1% (v/v) ammonium hydroxide (Sigma) and 1% (v/v) Triton X-100 (Sigma) in distilled water] for 2 days. After washing in distilled water, the derived ECM was digested with pepsin (1 mg/ml; Sigma) in HCl (0.01 N) for 2 days at room temperature until visible ECM particle disappeared. To make hydrogels, the ECM solution was mixed with 10 × PBS, dilute to the desired final concentration (20 mg/ml) with ice-cold distilled water and adjust to pH7 by NaOH (1 M). Finally, the ECM solution was mixed with collagen solution (4 mg/ml; BD biosciences) 10:1 (v/v) and solidified at 37°C. Cells were added at this stage prior solidification. Invadopodia length was measured and quantified with ImageJ.

### Colony Formation Assay

Radiation was performed with a^137^Cs irradiator (Shepherd). A total of 40 000 cells of neurosphere lines in 1 ml of neuro stem cell (NSA) media12 was radiated, mixed with 3 ml of 1.5% methylcellulose and 1 ml containing 10 000 cells per well was plated in triplicate in 12 well low attachment plate. Cells were fed every other day. Assays were done ≥3 times with individual samples in triplicate. Sphere formation was monitored and scored using GelCount (Oxford Optronix) after 10–12 days.

### Reverse phase Protein Array (RPPA)

The analysis of RPPA data was performed according to the protocol from the M.D. Anderson Cancer Center,^18^ on 913-GSCs and GBM1 cells expressing shGFP or shMT1-MMP. Relative protein levels for each sample were determined by interpolation of each dilution curves from the “standard curve” (supercurve) of the slide (antibody). Supercurve is constructed by a script in R written by the RPPA core facility. These values are defined as Supercurve Log_2_ value. All the data points were normalized for protein loading and transformed to linear value, designated as “Normalized Linear”, which was transformed to Log_2_ value, and then median-centered for further analysis. Median-Centered values were centered by subtracting the median of all samples in a given protein. All the above-mentioned procedures were performed by the RPPA core facility. Normalized values for each protein were reported as fold change of shMT1-MMP versus shGFP controls. Values are the mean of duplicate samples and of the two GSC lines.

### Western Blotting

Cell seeding (1 × 10^6^ cells per T25 culture flask), collection of protein and Western blot methods were as previously described.^11,19^ Membranes were probed with the following antibodies: anti-MT1-MMP (ab53712, Abcam); anti-MMP2 (clone EPR1184, Abcam); anti γH2AX (clone JBC301, EMD Millipore); anti-p-ChK1 (clone 133D3), anti p-ChK2 (clone C13C1), both from Cell Signaling Technology; anti H2AX (Abcam, ab20669), anti ChK1 (clone 2G1D5, Cell Signaling Technology), anti ChK2 (clone D9C6, Cell Signaling Technology); β-actin (clone C4) and GAPDH (clone 0411), both from Santa Cruz Biotechnology.

### Immunofluorescence

Cells were fixed in 3% formaldehyde and stained using standard procedures: γH2AX antibody (clone JBC301, EMD Millipore), secondary Alexa-Fluor 594 anti-mouse (A11032; Invitrogen, 1:500). Slides were mounted in Vectashield with DAPI (Vector Laboratories). Immunofluorescence was observed at ×40, foci were counted using ImageJ from at least 50 nuclei in five fields per slide, in triplicate, for each condition.

### Comet Assay

Comet assays were performed as previously described.^11^ Briefly, 1 × 10^6^ cells were irradiated or left untreated, then cultured for up to 24 h. Spheres were broken to single cells using accutase, counted, and the number of cells normalized among samples. 5 μl of each cell suspension was mixed with pre-melted low melting agarose and plated on glass slides provided in the kit. After solidification at 4°C, slides were immersed in cold lysis buffer. Electrophoresis was carried out at 21 V for 45 min using neutral electrophoresis buffer (1 × TBE). Slides were washed and then fixed in ethanol (70%) followed by drying at 37°C overnight. Slides were then stained with Sybr green DNA stain (1:10,000). Comets were imaged at ×10 magnification. Comet analysis was done using Comet Score (TriTek). A minimum 50 comets were included per condition.

### DNA Fiber Assay

DNA Fiber Assay was performed as previously described.^11,19^ To determine RF speed, cells were pulse-labeled with 250 μmol/l CldU for 30 minutes followed by a second pulse with 50 μmol/l IdU (Sigma) for another 30 min. For RF restart, cells were pulsed with 50 mol/l IdU for 40 min, followed by treatment with hydroxyurea to stall replication, then pulsed-labeled 250 μmol/l CldU (Sigma) for 40 min. Cells were then lysed [0.5% SDS, 200 mmol/l Tris-HCl (pH 7.4), 50 mmol/l EDTA] and dropped and spread onto an uncoated glass slide and let dry. DNA spreads were fixed with a 3:1 solution of methanol-acetic acid for 10 min, let dry and then placed in 70% ethanol at 4°C for 1 h. DNA was denatured with 2.5 mol/l HCl for 30 min at 37°C. Slides were blocked in 1% BSA and then incubated with mouse anti-BrdU antibody (BD Biosciences) and rat anti-CldU antibody (Abcam). Alexa Fluor 594, or Alexa Fluor 488 (Thermo Fisher Scientific) secondary antibodies were used. DNA fibers were viewed at ×100 magnification on a Keyence BZ-X800 microscope. Signals were measured using ImageJ as previously described.^11,19^

### Tumor Formation Assay

Animal studies were performed in accordance with University of Miami institutional guidelines. Eight-week-old female nude mice (Charles River) were injected intracranially with luciferase-expressing 913-GSC neurospheres with or without shGFP and shMT1-MMP stable expression, into the right cerebral cortex at a depth of 3 mm. Tumor growth was monitored twice weekly and quantified using bioluminescent imaging (BLI). Signal intensity was measured as photon counts within a Region Of Interest (ROI). One day post inoculation, mice were equally distributed into groups of treatment so that each group contained mice with similar tumor burden, measured by BLI. Mice that received *(R)*-ND336, were given the drug subcutaneously 5 days prior, on the day of, and 5 days post radiation at 25mg/kg daily. The treatment was then continued thrice weekly for an additional two months. The radiation groups received one single 12 Gy dose (equivalent to a BED—Biologically Effective Dose—between 26 and 40 based on a *a*/*b* value for gliomas ranging from 5 to 10^20^), using an XRad 320 cabinet irradiator to focally irradiate the right brain hemisphere with lead shielding to protect all other sites. Animal appearance, behavior, and weight were monitored to evaluate tumor progression as per a University of Miami approved IACUC protocol.

### Pharmacokinetics and Brain Penetration of (R)-ND-336

Female C57Bl6/J mice (6–8 weeks old, Envigo, *n* = 2 mice per time point) were administered a 10 mg/kg single subcutaneous dose of (*R*)-ND-336. At 1, 2, 4, and 8 h, the mice were sacrificed and terminal blood was collected by cardiac puncture and centrifuged to collect plasma. After transcardial perfusion with saline, the brains were harvested and immediately flash frozen in liquid nitrogen. Plasma and brain samples were stored at −80°C until analysis. Plasma was mixed with two volumes of internal standard in acetonitrile and centrifuged. Brain samples were weighed and homogenized in aqueous acetonitrile containing internal standard, followed by centrifugation. The plasma and brain supernatants were analyzed by ultraperformance liquid chromatography with electrospray ionization in the positive mode for the transition *m/z* 319 → 182 for (*R*)-ND-336 and *m/z* 300 → 93 for the internal standard. A Kinetex 2.6 µm, 2.1 mm i.d. × 75 mm length C18 column (Phenomenex) was used. The flow rate was 0.4 ml/min at 10% water/90% acetonitrile for 2 min, 8 min linear gradient to 90% acetonitrile/10% water. Calibration curves were prepared by spiking blank mouse plasma and blank mouse brain with various concentrations of (*R*)-ND-336. Peak area ratios relative to the internal standard and linear regression parameters were used for quantification. This study was conducted with approval and oversight by the IACUC at the University of Notre Dame.

## Results

### MT1-MMP Expression and Activity are Increased in GBM and Correlate with Patient Outcome

MT1-MMP has been shown to contribute to the aggressiveness of several cancers, including GBM.^8^ Here we sought to further characterize the importance of MT1-MMP in GBM to determine if targeting the protein might have clinical utility.

**Figure 1.**
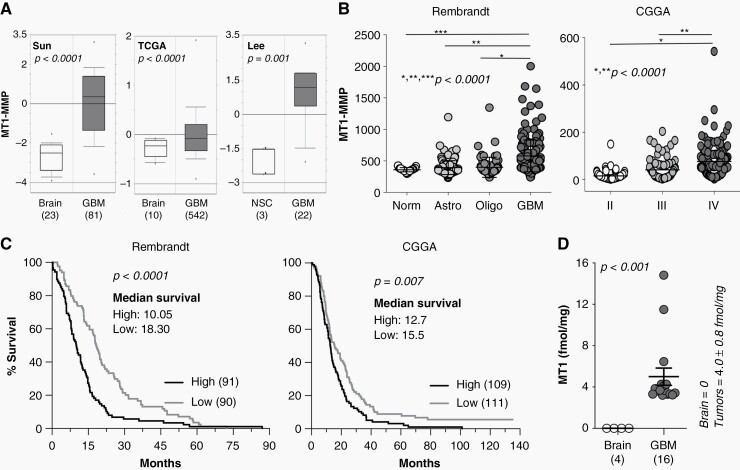
MT1-MMP is expressed and active in GBM and inversely correlated with patient survival. (A) Oncomine data comparing mRNA expression of *MMP14* between normal tissue and GBM in three datasets (NSC = neural stem cells). (B) MT1-MMP expression in progressively aggressive gliomas (Rembrandt and CGGA databases). (C) Kaplan–Meier plots of GBM from Rembrandt and CGGA, showing expression of MT1-MMP relative to patients’ overall survival. Data were obtained from GlioVis (http://gliovis.bioinfo.cnio.es/) and curves built with Prism9 GraphPad. (D) MT1-MMP enzyme activity in normal brain (*n* = 4) versus GBM (*n* = 16).

First, we interrogated publicly available data sets from the Oncomine database,^21^ which revealed that *MMP14* (the gene for MT1-MMP) expression is significantly increased in GBM versus normal (Figure 1A). These data were further confirmed by comparing the expression levels of MT1-MMP mRNA from patient derived GSCs collected from 28 GBM tumors, both primary and recurrent (R), to control, unaffected brain. The data showed 5 to 150-fold increase in MT1-MMP expression in the GSCs (Supplementary Figure 1A). Data from the Rembrandt and CGGA databases also revealed that the expression levels of MT1-MMP increase with glioma grade, being the highest in GBM (WHO grade IV) (Figure 1B). We then assessed the prognostic value of MT1-MMP expression and found the expression to be inversely associated with overall patient outcome in several datasets (Figure 1C, and Supplementary Figure 3).

To strengthen these findings, we sought to determine whether MT1-MMP activity was also increased in GBM. It is in fact only the active, catalytically competent protease that exerts its function. pro-MT1-MMP and TIMP-associated MT1-MMP, though assessable by immuno-histochemistry, western blotting and ELISA, are catalytically inactive, and generally indistinguishable from active MT1-MMP. To measure activity, we used a novel methodology coupling an affinity resin with proteomics^15^ (Supplementary Figure 4). The resin binds exclusively to the active forms of MMPs through accessing the active site, which is blocked in pro-MMPs and TIMP-MMPs. We obtained 16 primary GBM and 4 normal brain samples from surgical resections and measured MT1-MMP activity. We observed that all primary GBM tumors demonstrated higher MT1-MMP activity compared to normal brain, where activity was below the level of detection (Figure 1D).

Collectively, these data imply MT1-MMP is likely to play a role in the pathogenesis of GBM and may represent a novel target involved in GBM progression and therapeutic response.

### MT1-MMP Controls Glioma Stem-Like Cell Invasion Via MMP2

**Figure 2.**
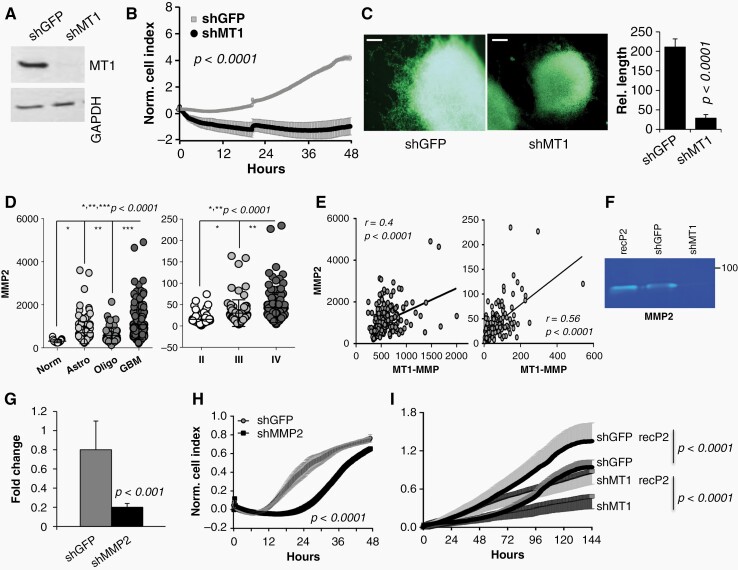
MT1-MMP mediates GBM invasion through MMP2. (A) MT1-MMP expression in 913-GSC upon shMT1-MMP transduction. (B) invasion of 913 through a matrigel-HA matrix (XCELLigence). (C) Invasion of 913-GSCs expressing shGFP or shMT1-MMP through a 3D brain derived matrix. Average length of invadopodia is shown. Scale bar, 50 µm. 100 spheroids per field were measured in 10 fields per slides (*n* = 3 slides). Experiments were repeated three times. (D) MMP2 expression in progressively aggressive gliomas (Rembrandt and CGGA databases). (E) Correlation between MMP2 and MT1-MMP in CGGA and Rembrandt, respectively. (F) zymogram of shGFP and shMT1-MMP expressing cells. Recombinant MMP2 was used as positive control. (G) Levels of MMP2 in 913-GSCs upon RNAi mediated MMP2 inhibition. (H) Invasion of the cells in G through a Matrigel/HA matrix (XCELLigence). (I) Invasion through a Matrigel/HA matrix of the cells in A with or without addition of recombinant MMP2 (10 ng/ml). Experiments were repeated three times. *P* values, calculated by the student’s *t* test, are shown.

Invasion through normal brain is a key determinant of the high recurrence rates of GBM, with multiple studies demonstrating that 78–95% of GBM recurrences occur within the 2 cm margin outside the MRI enhancement.^22,23^ MT1-MMP is a critical player in invasion and metastases in several cancers. For example, we have previously demonstrated that blockade of MT1-MMP in melanoma inhibits invasion as well as metastases in vivo.^13,24^ Given the high expression and activity of MT1-MMP in GBM patient samples, we determined whether MT1-MMP is required by GSCs to invade. MT1-MMP was inhibited by RNAi^11,25^ in 913-GSCs and GBM1 neurospheres (Figure 2A, and SupplementaryFigure 5A, respectively). Invasion was then assessed by xCELLigence, which measures cell invasion rates continuously. Inhibition of MT1-MMP significantly reduces the invasion capacity of GSCs through a Matrigel/HA matrix (Figure 2B, and Supplementary Figure 5B). Further, to address the invasive behavior of GSCs in a more physiological environment, we derived a 3D decelluarized mouse brain matrix,^16,17^ in which GSC neurospheres were embedded (Figure 2C). Also in this medium, inhibition of MT1-MMP significantly hampered GSC invasion, as indicated by the reduced length of invadopodia in cells expressing shMT1-MMP. These data were further validated using a second shRNA in 913-GSCs and in another GSC line (821-GSCs) (Supplementary Figure 5C, D).

**Figure 3.**
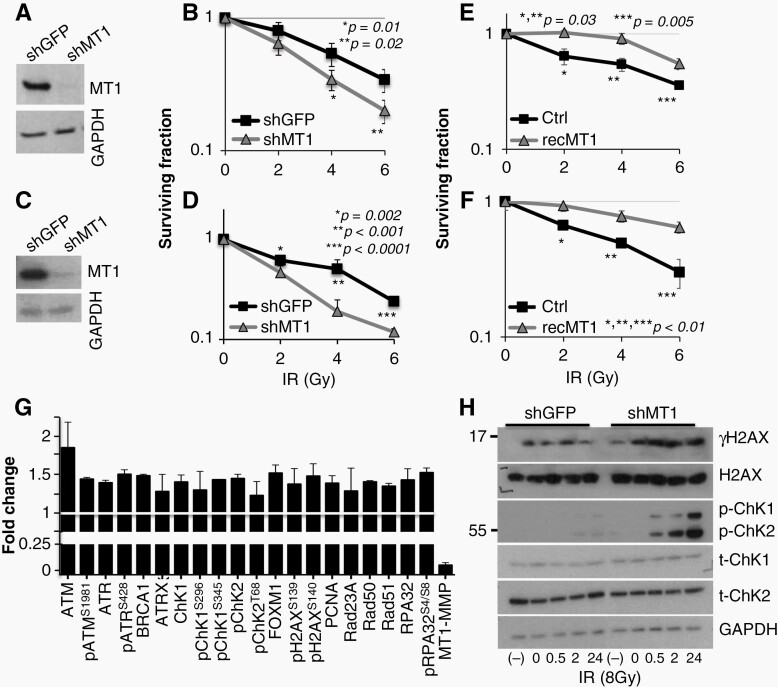
MT1-MMP mediates radiation response. knock down of MT1-MMP in 913-GSCs (A) and 821-GSCs (C). Clonogenic assay of 913-GSCs (B) and 821-GSCs (D) expressing shGFP or shMT1-MMP and exposed to increasing radiation doses (Gy). (E, F) Clonogenic assay of 913 and 821-GSCs, respectively, treated with recombinant MT1-MMP (20 ng/ml). *P* values are shown. Experiments were repeated three times. (G) Mean fold increase of DDR proteins assessed by RPPA in 913-GSC and GBM1. (H) time course for γH2AX, p-ChK1 and 2, after one 6Gy IR dose (913-GSCs). (−): baseline, no radiation; 0–24 h: recovery after radiation.

**Figure 4.**
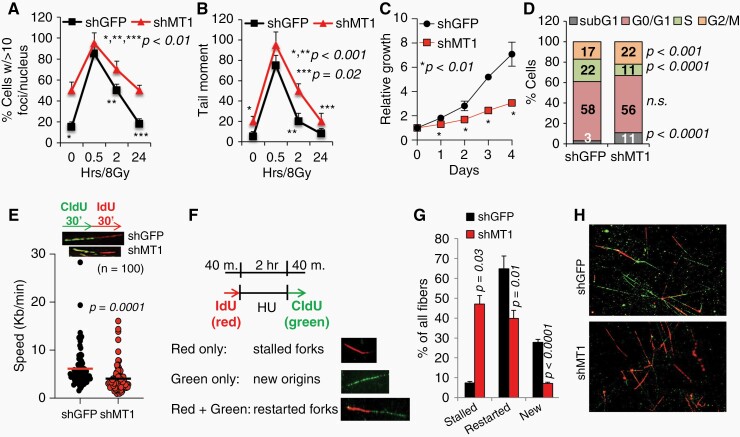
Inhibition of MT1-MMP increases DNA damage, reduces growth and causes replication fork stress. (A) γH2AX foci in shGFP and shMT1-MMP 913-GSCs expressing cells. At least 50 nuclei per field were counted, for a total of ten fields per slide (*n* = 3 slides per condition). (B) Neutral Comet Assay in shGFP and shMT1-MMP 913-GSCs expressing cells. (C) shGFP and shMT1-MMP expressing 913-GSCs in vitro growth. Experiments were repeated three times. The data are the mean among them. (D) Cell cycle profile of the cells in (C). (E) Replication fork speed measured in shGFP and shMT1-MMP expressing cells. At least 100 fibers per group were counted. Speed was calculated by dividing the length of IdU tracts by 30 min (time of IdU pulse). Length (um) per minute was converted to kb per minute. (F) Scheme of treatment. Red only fibers: stalled forks; Green only fibers: new origin; Red + Green: restarted forks after HU. (G) % of all fibers was calculated out of 200 DNA fibers counted in all groups. (H) Representative CldU and IdU stained DNA fibers. Data are the average of three independent experiments. *P* values, calculated by the Student’s *t* test, are shown.

MT1-MMP can directly process ECM components and can also promote invasion through the activation of effector proteases.^26^ MMP2 is directly activated by MT1-MMP^27^; and MMP2 has been associated with poor GBM patient outcome.^28^ We found that, similarly to MT1-MMP, the expression of MMP2 increases with disease progression in both the Rembrandt (Figure 2D, left panel) and CGGA databases (Figure 2D, right panel). Additionally, a significant correlation between MT1-MMP and MMP2 exists (Figure 2E, Rembrandt: right panel; CGGA: left panel), underlying their co-deregulation and potential cooperation in the disease. This was further corroborated by the observation that depletion of MT1-MMP causes reduced MMP2 activity, assessed by a gelatin zymography (Figure 2F). We therefore tested whether MMP2 was involved in GBM invasion and found that indeed, inhibition of MMP2 by a specific shRNA^24,29^ (Figure 2G) significantly decreases 913-GSC invasion (Figure 2H). Treatment of MT1-MMP depleted cells with recombinant MMP2 (recP2), restored the cells invasive capacity to the levels of controls and increased the invasive abilities of control cells (Figure 2I), further indicating MMP2 acts downstream of MT1-MMP in mediating invasion of GSCs.

### Inhibition of MT1-MMP Sensitizes Patient Derived GSCs to Ionizing Radiation by Increasing Endogenous DNA Damage

We have previously shown in a breast cancer model^11^ that MT1-MMP protects cancer cells from radiation and chemotherapy, and that inhibition of MT1-MMP is sufficient to sensitize breast cancer to these treatments. Given that radiation is standard of care after surgery in GBM patients, we asked whether a similar mechanism was in place for GBM. 913-GSCs and 821-GSCs^12,30^ expressing an shRNA control (shGFP) or shMT1-MMP (Figure 3A, C, respectively) were subjected to several increasing doses of ionizing radiation (IR), and survival was evaluated by a standard clonogenic assay. MT1-MMP depleted cells (Figure 3B-913, D-821) were significantly more sensitive to IR, supporting a role of MT1-MMP in regulating DNA damage. Similar data were obtained in both GSCs when a second shRNA was used, further confirming the data and excluding off target effects of the RNAi sequences (Supplementary Figure 6). On the other hand, addition of recombinant active MT1-MMP protected both GSC lines from radiation (Figure 3E-913, F-821).

MMP2 is directly activated by MT1-MMP, functioning in most cases as its effector, as is the case in invasion of GSCs, and it has been previously shown to protect lung cancer cells from radiation.^31^ However, in our system, MMP2 depletion did not affect the response to radiation in either GSC line (Supplementary Figure 7), nor did it increase DNA damage, alone or after radiation, as indicated by unchanged γH2AX foci and tail moment from a comet assay (Supplementary Figure 16F–I), suggesting MT1-MMP controls DNA damage responses (DDR) independently of MMP2.

We therefore further characterized the DNA damage associated with MT1-MMP inhibition. Data from Reverse Phase Protein Array (RPPA) from two GSC lines expressing shGFP or shMT1-MMP demonstrated an average 1.5-fold increase of several DNA Repair Damage (DDR) proteins, both the total and phosphorylated (active) forms, in cells depleted of MT1-MMP (Figure 3G). To strengthen these data, 913-GSC expressing shGFP or shMT1-MMP, were subjected to 6Gy IR then the levels of γH2AX and ChK1 and ChK2 activation were measured by immunoblotting up to 24 h after radiation (Figure 3H). Interestingly, pChK1 and 2 signals were much stronger and continued to increase during the time course, in cells depleted of MT1-MMP compared to control cells. Similar results were observed in 821-GSCs as well (Supplementary Figure 8A). MT1-MMP depleted cells also demonstrated higher γH2AX at baseline [(−)] and maintained higher levels throughout.

This was further corroborated by quantifying γH2AX foci and by a neutral comet assay. Quantification of γH2AX foci revealed that 913-GSC neurospheres depleted of shMT1-MMP had a four-fold increase in endogenous γH2AX foci (Figure 4A, 10% vs >40% nuclei with >10 foci, respectively, and Supplementary Figure 9), and retained higher levels of γH2AX foci throughout. Also, in the comet assay, 913-GSC neurospheres depleted of MT1-MMP started out with greater DNA damage (2.8-fold increase in tail length of shMT1 vs shGFP) and accumulated slightly more damage upon IR with respect to shGFP expressing cells (Figure 4B). Both cells returned to their baseline DNA damage levels, which, for cells expressing shMT1-MMP, remain almost 3 times higher. Again, a similar trend was observed in the 821-GSC line (Supplementary Figure 8B–D). Thus, GSCs deprived of MT1-MMP accumulate endogenous DNA damage, which is likely to sensitize them further to damage stimuli (eg, radiation) by lowering the threshold of tolerable additional DNA damage, and explaining the higher rate of death observed upon radiation treatment.

### MT1-MMP Effects Cell Cycle and Fork Replication Stability

Given the increase in basal DSBs, we posited that MT1-MMP might be important in preventing the formation of DNA damage during replication, as replication stress is a major endogenous source of DSBs.^32,33^ To address this possibility in GBM, we first tested whether MT1-MMP would effect cell growth and cell cycle progression. Depletion of MT1-MMP reduced cell growth (Figure 4C) and was accompanied by a 50% reduction of cells in S phase, a 25% increase in cells in G2/M, and a slight but significant increase in subG1 cells (Figure 4D and Supplementary Figure 10A) in 913 GSCs. Similar data were obtained in 821-GSCs depleted of MT1-MMP (Supplementary Figure 10B–D). Accumulation in G2/M can occur when cells are experiencing slowing or stalling of replication forks, which result in the exposure of longer patches of single stranded DNA, ultimately leading to activation of ATR/ChK1. This is in accordance to the data in Figure 3H. ATR/ChK1 activation then inhibits the entry into mitosis to allow resolution of replication stress.^34^ If resolution does not occur, forks collapse into DSBs.^35–37^ To determine fork speed, cells were incubated with CldU (chloro-deoxyuridine) for 30 min then pulsed with IdU (Iodo-deoxyuridine) for the same time. The IdU tracts were measured, and the length divided by the IdU pulse time (30 min) and converted in kb/min using the conversion factor: 1 µm = 2.59 kb.^38^ MT1-MMP depleted cells demonstrated slower RF speed (Figure 4E). Subsequently, to determine restart after stalling, cells were incubated with IdU followed by the addition of Hydroxy Urea (HU) to deplete the nucleotide pool and stall replication forks. After HU removal, cells were labeled with CldU to quantify the ability to restart replication (Figure 4F–H). Cells depleted of MT1-MMP showed slower RFs speed as well as a six-fold more stalled forks, a 1.6-fold reduction in the number of restarted forks, and a four-fold reduction in fired new origins when compared to control cells. In support of these data, we found an increased in RPA1 foci in cell depleted of MT1-MMP which also appeared smaller and more distributed throughout the nucleus compared to control cells (Supplementary Figure 11). RPA1 is a major ssDNA-binding protein that protects ssDNA from degradation and that serves as scaffold for the recruitment of DNA damage response factors.^39^ This suggests an increase in exposed ssDNA in cells in which MT1-MMP was inhibited, in line with slower RF speed and defects in RF restart. These data indicate that cells depleted of MT1-MMP undergo replication stress represented by stalled replication forks. This is likely to be followed by persistent unrepaired DNA, with accumulation of DSBs.^37^ Higher, persistent endogenous DNA damage leads to higher sensitivity of cells to further genotoxic stresses.

### Inhibition of MT1-MMP Increases Survival and Radiation Response

**Figure 5.**
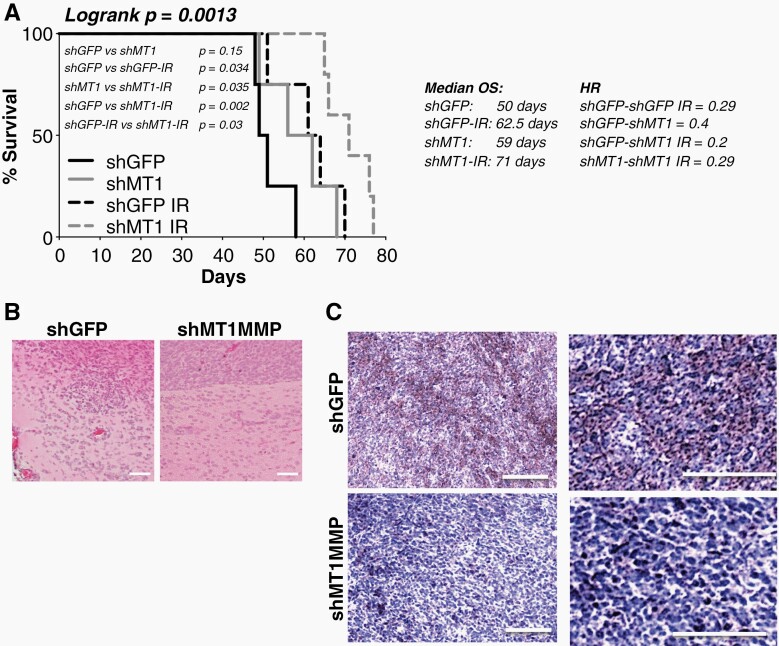
MT1-MMP inhibition synergizes with radiation improving survival. (A) 10^5^ 913-GSCs expressing luciferase, were inoculated stereotactically into the right basal ganglia of athymic nudes. Tumors were monitored twice weekly since inoculation. Kaplan–Meier curves were generated with Graph Pad Prism9. Logrank p values, median overall survival (OS) and Hazard Ratio (HR) are shown. (B) H&E staining in shGFP and shMT1-MMP tumors. Scale bars: 50 µm. (C) Mt1-MMP staining intensity in shGFP and shMT1 tumors. Scale bars: 100 µm.

Given the effects of MT1-MMP inhibition on cell growth and radiation response in vitro, we next determined if inhibition of the protease would improve survival and radiation response in vivo. 10^5^ luciferase-expressing shMT1-MMP or shGFP expressing 913-GSCs were injected stereotactically into the right basal ganglia of athymic nude mice. Tumor growth was monitored twice weekly and quantified using bioluminescent imaging (BLI). One day post inoculation, mice were equally distributed into four groups of treatment so that each group contained mice with comparable tumor burden, measured by BLI. Mice were then irradiated by one single dose of 12 Gy. Animals were monitored by BLI to record tumor growth/expansion and to assess their well-being. MT1-MMP inhibition increased survival slightly, albeit not significantly, and did improve response to IR by further extending survival compared to control (shGFP) tumors as well as irradiated shGFP tumors (Figure 5A), indicating that combining MT1-MMP inhibition with radiation results in better outcomes. Finally, MT1-MMP inhibition seemed to be associated with reduced invasion. Tumors in the shMT1-MMP group have more defined boundaries, with reduced numbers of invading cells compared to shGFP tumors which demonstrate disrupted tumor edges and invasive cells in the brain (Figure 5B). Also, the shMT1-MMP tumors retained the shRNA expression as shown by lower staining intensity compared to controls, which expressed MT1-MMP in patches throughout the tumor bulk (Figure 5C). These data confirm MT1-MMP plays a role in GBM invasion and protects GBM tumors from radiation.

#### The selective catalytic inhibitor (R)-ND336 sensitizes GBM tumors to radiation and extends survival.

**Figure 6.**
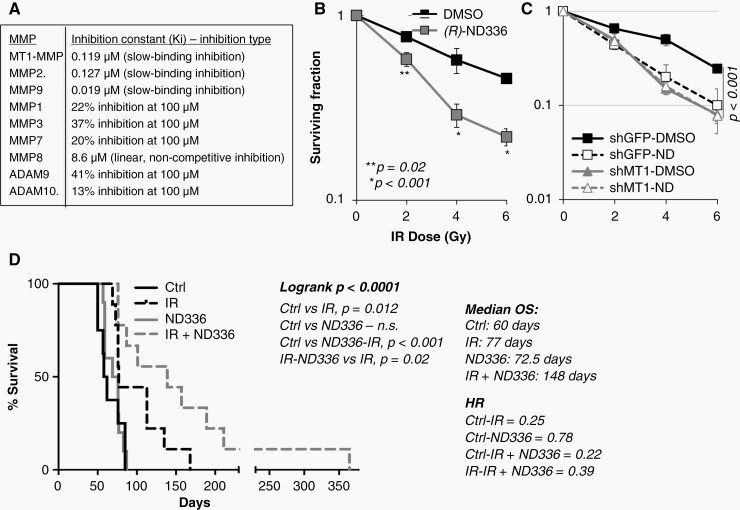
*(R)*-ND336 sensitizes to radiation both in vitro and in vivo. (A) *k*i values and type of inhibition (slow-binding and linear non-competitive) of (R)-ND336 for a variety of MMPs. (B) Clonogenic assay of 913-GSC cells treated with 0.30 μg/ml (R)-ND336 and subjected to increasing doses of radiation. Data are the mean of two independent experiments. *P* values, determine by the Student’s *t* test, are shown. (C) Clonogenic assay of 913-GSC cells treated with 0.30 μg/ml (R)-ND336 and expressing either shGFP or shMT1-MMP. (D) 7 × 10^4^ 913-GSCs expressing luciferase, were inoculated stereotactically into the right basal ganglia of athymic nudes. Tumors were monitored twice weekly since inoculation. (R)-ND336 was given daily at 25 mg/kg s.c., starting at 5 days prior, on the day of, and 5 days post radiation, to be then continued for 2 months twice weekly. Kaplan–Meier curves were generated with Graph Pad Prism9. Logrank *P* values, median overall survival (OS) and Hazard Ratio (HR) are shown.

—To address the potential clinical translation of MT1-MMP blockade, we assessed the efficacy of *(R)*-ND336, a novel, selective MMP catalytic inhibitor (Figure 6). *(R)*-ND336 has been successfully used for the treatment of diabetic foot ulcer (DFU)^15,40^ in preclinical models, and is anticipated to enter a phase I clinical trial for the treatment of DFUs in 2023. *(R)*-ND336 is a third generation selective MT1-MMP/MMP2/MMP9 inhibitor. Much like its analog ND322 we have previously used in a melanoma model of metastasis^24^ and therapy resistance^25^; and the prototypic SB-3CT,^41^*(R)*-ND336 is a slow-binding inhibitor of MT1-MMP and the gelatinases MMP2 and MMP9 (Figure 6A, inhibition constants—*ki*—and type of inhibition). Slow-binding inhibition results in a conformational change in the inhibitor-enzyme complex that is not easily reversed. Thus, slow-binding inhibition results in long residence times that confer sustained inhibitory duration and selectivity towards the targets. *(R)*-ND336 residence times for MT1-MMP, MMP2 and MMP9 are higher than those of TIMPs,^42^ the endogenous MMP inhibitors that have evolved for this purpose. Importantly, *(R)*-ND336 exhibits marginal to no inhibition of several other MMPs (Figure 6A), including MMP8, which has been shown to possess anti-tumor activity; and ADAM proteases whose inhibition was at the root of a musculoskeletal syndrome associated with pan-MMP inhibitors that contributed to the discontinuation of clinical trials of these drugs.^43^ Finally, *(R)*-ND336 was chosen for its ability to effectively cross the blood brain barrier (BBB). *(R)*-ND336 shows concentrations in brain similar to plasma throughout 8 h after a single subcutaneous dose of 10 mg/kg (Supplementary Figure 12).

We first tested *(R)*-ND336 in vitro to determine if it was able to sensitize GSCs to radiation. At a dose of 0.15 μM, *(R)*-ND336 was indeed capable of sensitizing 913-GSCs to several IR doses (Figure 6B). Concomitant siRNA mediated depletion of MT1-MMP did not produce additive effects (Figure 6C). Cell growth was also reduced but no additive effects were observed when combining the drug with RNAi against MT1-MMP (Supplementary Figure 13), indicating *(R)*-ND-336 effects GSC growth and radio-sensitization via inhibition of MT1-MMP. Finally, the inhibitor was also able to reduce invasion, both through a matrigel/HA matrix as well as a brain matrix (Supplementary Figure 14). Therefore, *(R)*-ND336 is an effective, stable, and selective inhibitor that effectively crosses the BBB.

To test *(R)*-ND336 efficacy in vivo, 7 × 10^4^ 913-GSCs were inoculated stereotactically into the brains of athymic nude mice. Tumor growth was monitored twice weekly by bioluminescent imaging (BLI). One day post inoculation, the mice were equally distributed into four groups of treatments so that each group contained mice with similar tumor burden. Mice were treated daily with 25 mg/kg *(R)*-ND336 s.c. for 5 days prior to a single dose of 12 Gy of X-rays^20^; and for an additional 5 days after irradiation in the IR*+ (R)*-ND336 group, while the *(R)*-ND336 only group received the drug regimen. We chose this regimen to ensure MT1-MMP inhibition at the time of irradiation and afterwards, during DNA repair. Interestingly, although *(R)*-ND336 alone did not extend survival, it did synergize with radiation, almost doubling the median survival of mice treated with radiation alone (Figure 6D; IR only: 77 days; IR+ *(R)*-ND336: 148 days). Of note, the last surviving animal, still showed presence of tumor in the brain prior to euthanasia at day 365 post inoculation (Supplementary Figure 15).

*(R)*-ND336 is also an inhibitor of MMP2 and MMP9. While we have shown that radio-sensitization is MT1-MMP dependent, and invasion is a function of MMP2 downstream of MT1-MMP (Figure 3), we wanted to determine the possible contribution of MMP9, since MMP9 has been associated with poorer GBM patient outcome.^28,44^ Interestingly, while MMP9 inhibition (Supplementary Figure 16A) reduced invasion through a Matrigel/HA matrix (Supplementary Figure 16B), it did not affect radio-sensitization (Supplementary Figure 16C–I), indicating MT1-MMP directly promotes resistance to IR and that *(R)*-ND336 radio-sensitization is via MT1-MMP.

## Discussion

Glioblastoma multiforme (GBM) is the most common malignant brain tumor, comprising 54% of all gliomas and 14.6% of CNS tumors.^1^ Treatment for GBM has been almost unchanged for the past 20 years. Recent introduction of immunotherapy has shown little success in clinical trials, mainly because GBM is typically a “cold”, non-inflamed tumor. Indeed, GBM tends to induce an immunosuppressed TME enriched in immunosuppressive chemokines (eg, TGFβ, IL10, IL6) secreted by tumor cells, microglia and tumor-associated macrophages (TAMs). This results in inhibition of both the innate and adaptive immune systems, key elements in the success of immune checkpoint inhibitors such as anti-PD1.^45^

Here we demonstrate that targeting the membrane bound matrix metalloproteinase MT1-MMP can be an effective avenue to counteract GBM invasion and resistance to therapy. By using patient derived GSCs we demonstrate that MT1-MMP mediates GBM invasion both in vitro and in vivo, via its effector protease MMP2; whereas the radiation response is a function of MT1-MMPs ability to control replication fork stability.

Similar results were observed in breast cancer cells, in which depletion of MT1-MMP also led to replication fork stalling and collapse.^11^ These data suggest the involvement of MT1-MMP in DNA repair may be generalized to tumors expressing the protein rather than tissue of origin, thus making MT1-MMP an intriguing target in invasive tumors undergoing radio- and chemo-therapy.

Of note, we utilize for the first time in a GBM orthotopic model, *(R)*-ND336, a novel selective MMP inhibitor, which has shown efficacy in the treatment of diabetic foot ulcer and for which IND-enabling studies are ongoing. The strengths of this compound are its selectivity, water solubility,^15^ stability in vivo, and its ability to cross the blood brain barrier (BBB).

It has become more and more clear that there is a distinction between the BBB and the brain tumor barrier (BTB), which is highly heterogeneous and characterized by a non-uniform permeability and active efflux of molecules. For example, studies with an intracranial glioma model have shown that doxorubicin distribution is heterogeneous, with higher concentration of drug in the tumor core and less in the surrounding brain stroma. Also, in general, GBM tumors tend to disrupt the BBB as they progress, whereas oligodendroglioma models show less disruption. This heterogeneous drug perfusion within the tumor microenvironment and among tumors and the heterogeneous permeability to small and large molecules contributes to suboptimal drug accumulation in brain tumors (reviewed in^46^).

Although we did not directly test the efficacy of *(R)*-ND336 against the targets in vivo, *(R)*-ND336 shows almost 100% penetration in the normal, unaffected brain, thus it is likely to reach effectively the tumor cells. Indeed, we observed doubling of survival in mice treated with the drug and radiation versus radiation alone, which is better even that the results obtained with genetic inhibition of MT1-MMP.

It is possible that *(R)*-ND336, which is given systemically, may affect not only the tumor but also the tumor microenvironment (TME). For example, it has been shown that MT1-MMP inhibits cytotoxic T-cells and promotes an M2 phenotype of Tumor Associated Macrophages (TAMs) in breast cancer.^47^ The GBM TME contains mainly tumor-associated microglia and TAMs, which constitute up to 30% of the total tumor.^48^ M2 TAMs, which are considered pro-tumorigenic due to their anti-inflammatory characteristics, have been shown to promote tumorigenesis and are associated to radiation resistance in GBM.^49^ TAMs also upregulate MT1-MMP and MMP2 once exposed to tumor cells, which contributes to GBM aggressiveness.^50^ Thus, blockade of stromal MT1-MMP may also contribute to the anti-tumor effects of *(R)*-ND336. Additionally, the pro-inflammatory effects of MT1-MMP blockade observed in breast cancer suggests this strategy could potentially also improve immunotherapy in GBM.

In conclusion, we demonstrate MT1-MMP is highly expressed and active in GBM and associated with poor patient outcome. We also show that MT1-MMP is associated with resistance to radiation through its role in replication fork stability. Importantly, we show that targeting MT1-MMP by the novel selective small molecule *(R)*-ND336 effectively extends survival in preclinical GBM models. Future studies will address how the combination *(R)*-ND336-radiation compares to temozolomide (TMZ)-radiation as a potentially less toxic alternative which could also be utilized in TMZ resistant tumors as well as in patients that relapse on the current standard of care.

## Supplementary Material

vdac147_suppl_Supplementary_Figures
