# Supplementary material for: Targeting extracellular matrix remodeling sensitizes glioblastoma to ionizing radiation
Source: Neurooncol Adv. 2022 Sep 10;4(1):vdac147. doi: 10.1093/noajnl/vdac147 (PMC9536293; doi:10.1093/noajnl/vdac147)
Supplement: vdac147_suppl_Supplementary_Figures [file vdac147_suppl_supplementary_figures.pdf]

A

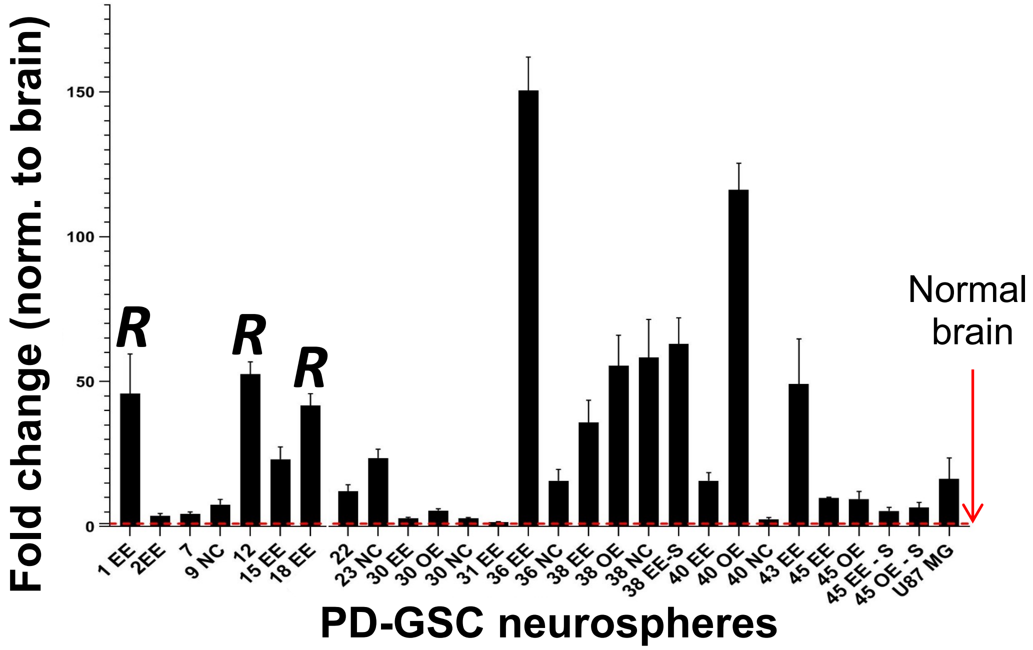

B

	Patient Information												
Cell Line	Sex	Age	Ethnicity	Previous different cancer?(Y/N)	If Y, what type of cancer?	Ever received Chemo? (Y/N)	Ever received Radiation? (Y/N)	GBM Status	Newly diagnosed multifocal? (Y/N)	After resection, recurrent in different location? (Y/N)	IDH status	MGMT status	Regions
GBM1	M	70	Hispanic	N	-	Y	Y	Recurrent	N	N	Negative	Positive	EE/OE/NC
GBM2	F	81	N/A	N		N	N	Newly Diag	N		N/A	Negative	EE
GBM7	F	58	White	N		N	N	Newly Diag	N		Negative	Negative	-
GBM9	F	37	N/A	N		Y	Y	grade 3 astrocytoma	N	N	Positive	N/A	NC
GBM12	M	68		Y	Thyroid cancer and smoldering myeloma	Y	Y	Recurrent	N	N	N/A	Negative	-
GBM15	F	33	White	N		N	N	Newly Diag	N		N/A	N/A	EE
GBM18	F	73	White	N	-	Y	Y	Recurrent	N		Negative	Positive	EE/OE/NC
GBM22	M	46	Asian	N	-	N	N	Newly Diag	N	Y	Negative	Negative	Mix
GBM23	F	74	White	Y	Melanoma and basal cell carcinoma	Y	Y	Recurrent	N	Y	Positive	Positive	EE/OE/NC
GBM30	F	60	Hispanic	N	-	N	N	Newly Diag	N	N/A			EE/OE/NC
GBM31	M	47	African-American	N	-	N	N	Newly Diag	Y	N/A	Negative	Negative	EE/OE/NC
GBM36	M	80	Hispanic	Y	prostate carcinoma	N	Y	Newly Diag	N	N	Negative	Negative	EE/OE/NC
GBM38	M	77	Hispanic	N	-	N	N	Newly Diag	N		Positive	Positive	EE/OE/NC
GBM40	M	59	White	N	-	N	N	Newly Diag	N	N	Negative	Negative	EE/OE/NC
GBM43	M	71	White	Y	prostate carcinoma	N	N	Newly Diag			Negative	Negative	EE/OE/NC
GBM45	F	78	Hispanic	N	-	N	N	Newly Diag			Negative	Positive	EE/OE/NC

Suppl. Fig. 1: A) RT-PCR for MT1-MMP in GSC lines, normalized to GAPDH. Fold change was calculated with respect to MT1-MMP levels of normal brain (solid red line). **B)** GSCs derived from patients at the Neurological Surgery Clinic at The University of Miami. EE: enhancing edge; OE: outside the enhancing edge; NC: necrotic center.

Suppl. Figure 2. Peptides used for the affinity resin/proteomics quantification of active MMPs in human GBM samples and control human brains[a]

Protein	Peptide sequence	Q1 precursor ion *m/z*	Q3 product ion *m/z*
**MT1-MMP**	**VGEYATYEAIR**	**636.32 [M+H]$^{2+}$**	**651.35 [M+H]+ y5**<br>**752.39 [M+H]$^{+}$ y6**<br>**823.43 [M+H]$^{+}$ y7**
	VWESATPLR	529.79 [M+H]$^{2+}$	644.37 [M+H]$^{+}$ y6<br>773.42 [M+H]$^{+}$ y7<br>959.49 [M+H]$^{+}$ y8
	EVPYAYIR	505.77 [M+H]$^{2+}$	685.37 [M+H]$^{+}$ y5<br>782.42 [M+H]$^{+}$ y6<br>881.49 [M+H]$^{+}$ y7
**Internal standard (yeast enolase)**	TAGIQIVADDLTVTNPK	878.48 [M+H]$^{2+}$	772.46 [M+H]$^{+}$ y7<br>1002.51 [M+H]$^{+}$ y9<br>1172.62 [M+H]$^{+}$ y11
	VNQIGTLSESIK	644.86 [M+H]$^{2+}$	676.39 [M+H]$^{+}$ y6<br>777.44 [M+H]$^{+}$ y7<br>1075.60 [M+H]$^{+}$ y10
	**NVNDVIAPAFVK**	**643.86 [M+H]$^{2+}$**	**561.34 [M+H]$^{+}$ y5**<br>**632.38 [M+H]$^{+}$ y6**<br>**844.53 [M+H]$^{+}$ y8**

[a] For quantification, three peptides per proteinase were used. In Q1 (first quadrupole), precursor ions were selected, then fragmented in Q2, and the specified product ions were monitored in Q3. In bold are the fragments used for quantification.

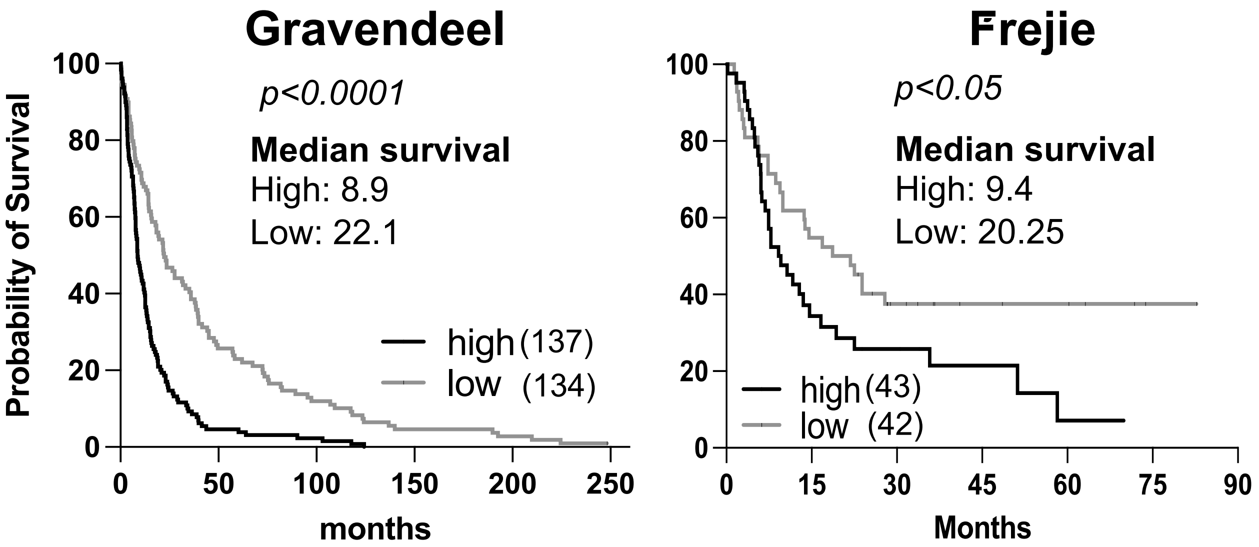

Suppl. Fig. 3: survival curves based on MT1-MMP expression levels from the Gravendeel and Frejie GBM data sets. Number of patients are in parenthesis. Data were obtained from GlioVis (http://gliovis.bioinfo.cnio.es/).

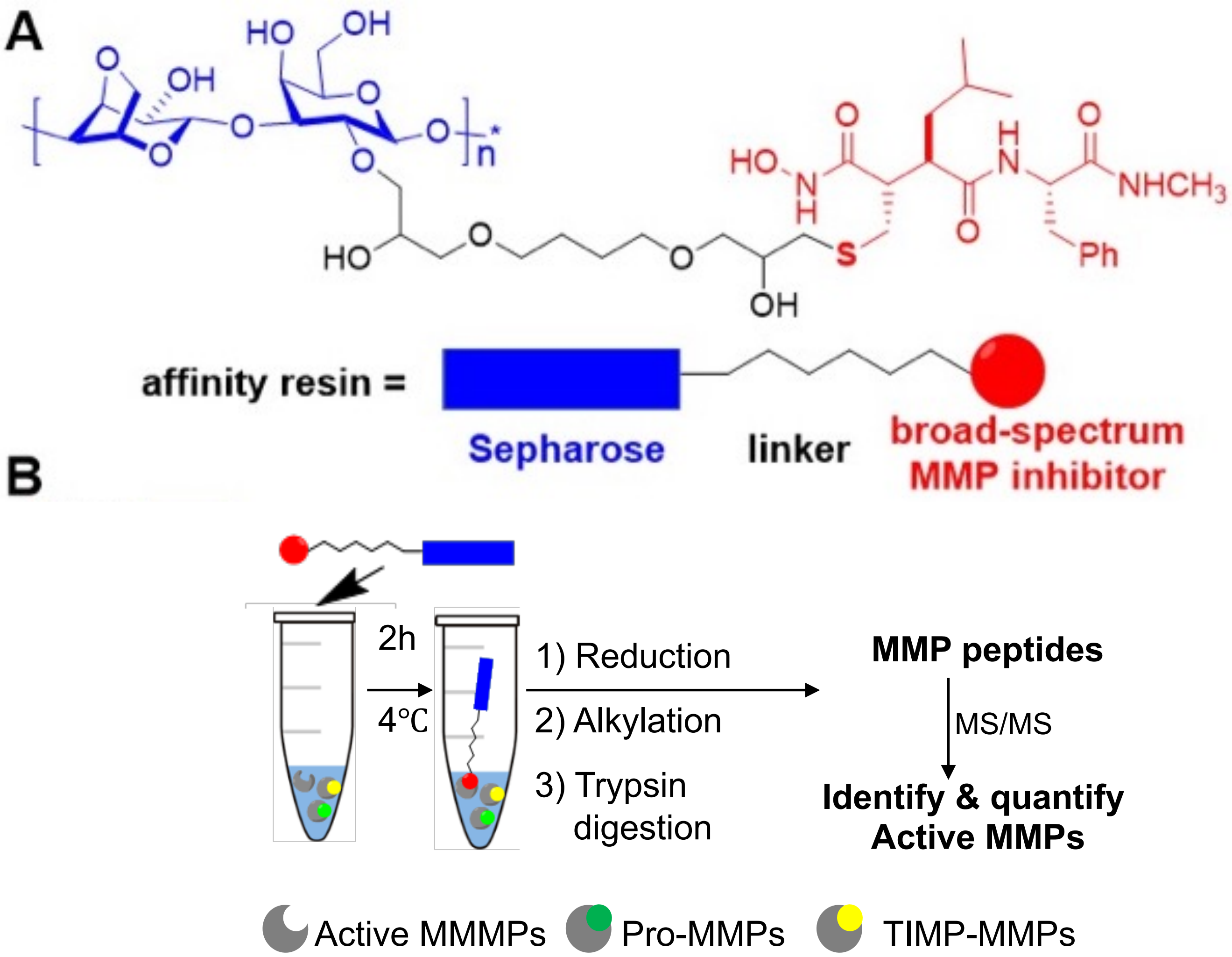

Suppl. Fig. 4: Scheme of workflow to measure activity of MT1-MMP (and other MMPs) by the novel affinity resin coupled with Mass Spectrometry.

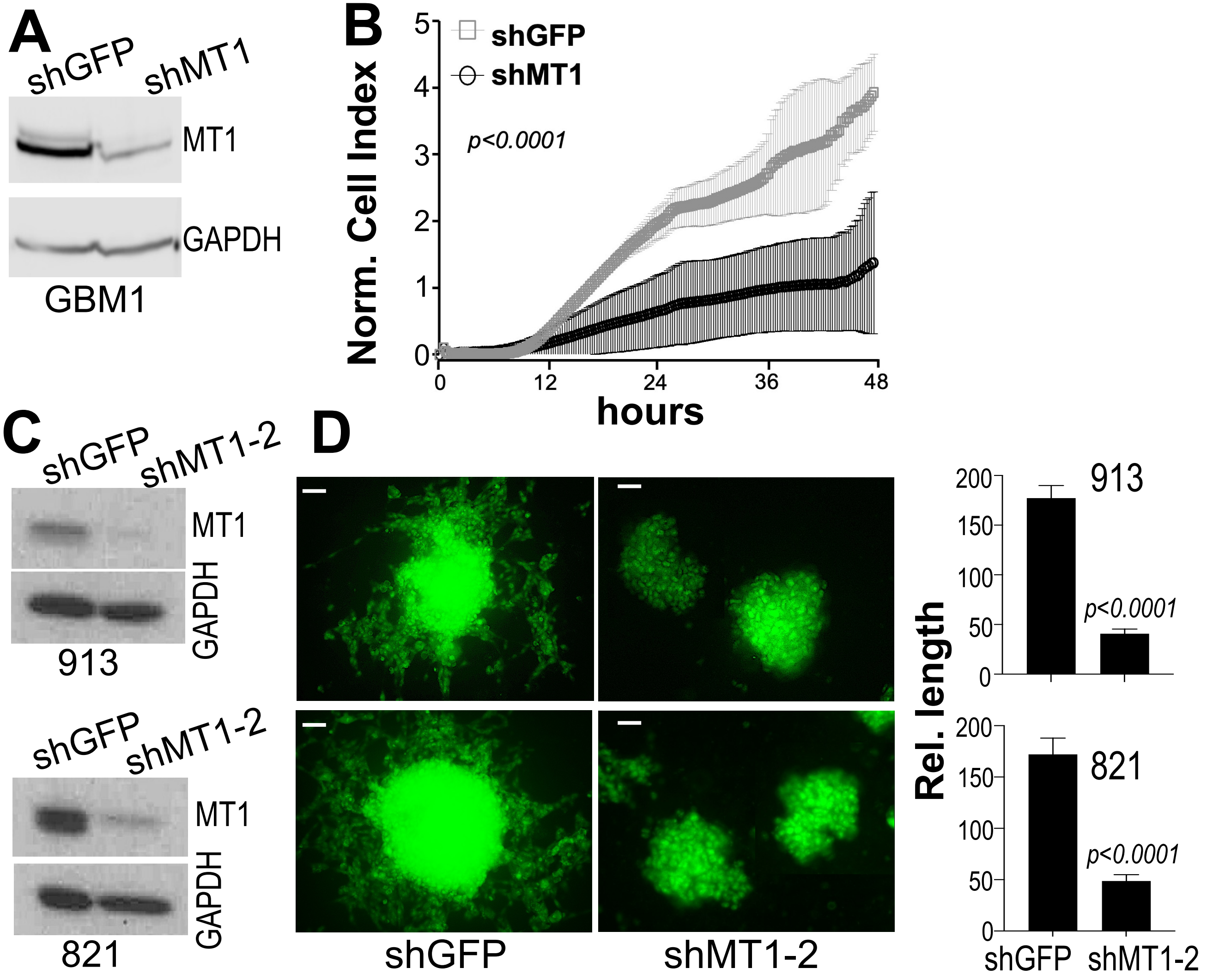

Suppl. Fig. 5: A) MT1-MMP expression in GBM1 GSCs expressing shGFP or shMT1-MMP (clone 1). **B)** Invasion through a Matrigel/HA matrix (XCELLigence) of the cells in A. **C)** MT1-MMP expression in 913- and 821-GSCs expressing shMT1-MMP-2 (Signa Mission # TRCN0000050856). **D)** invasion of the cells in C, through a brain matrix. Quantification was done using ImageJ. Scale bar=50um. n=50 spheroids per group.

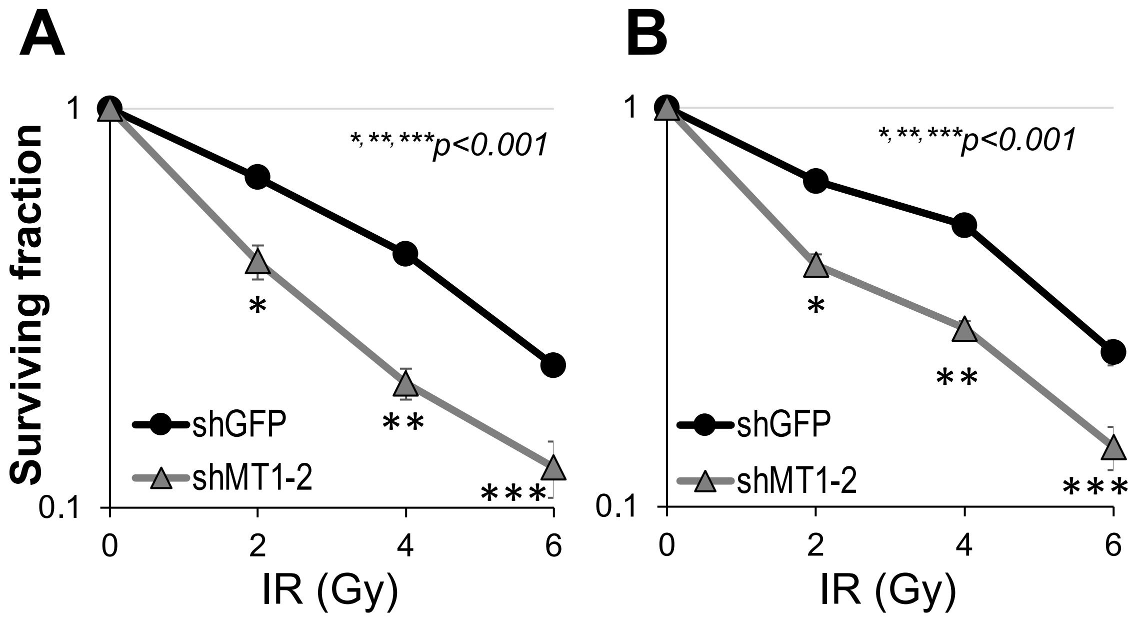

Suppl. Fig. 6: A, B) Clonogenic assay of 931 (A) and 821 (B) GSCs cells expressing shGFP or shMT1-MMP-2 (Sigma mission # TRCN0000050856).

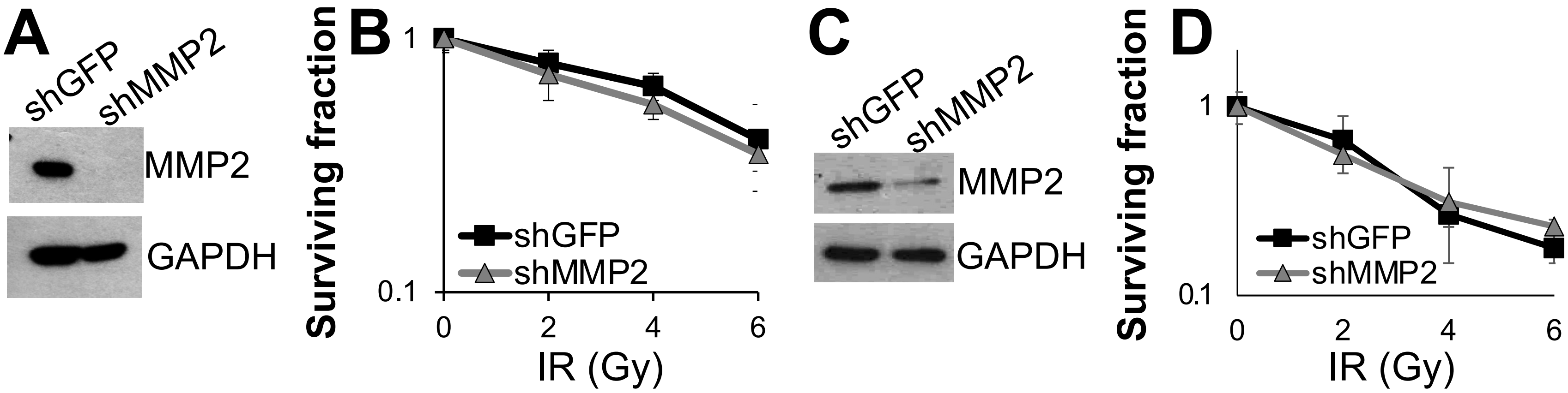

Suppl. Fig. 7: MMP2 inhibition does not sensitize to radiation. 913-GSC (**A, B**) and 821-GSC (**C, D**) cells expressing shGFP or shMMP2 and then treated with escalating doses of IR.

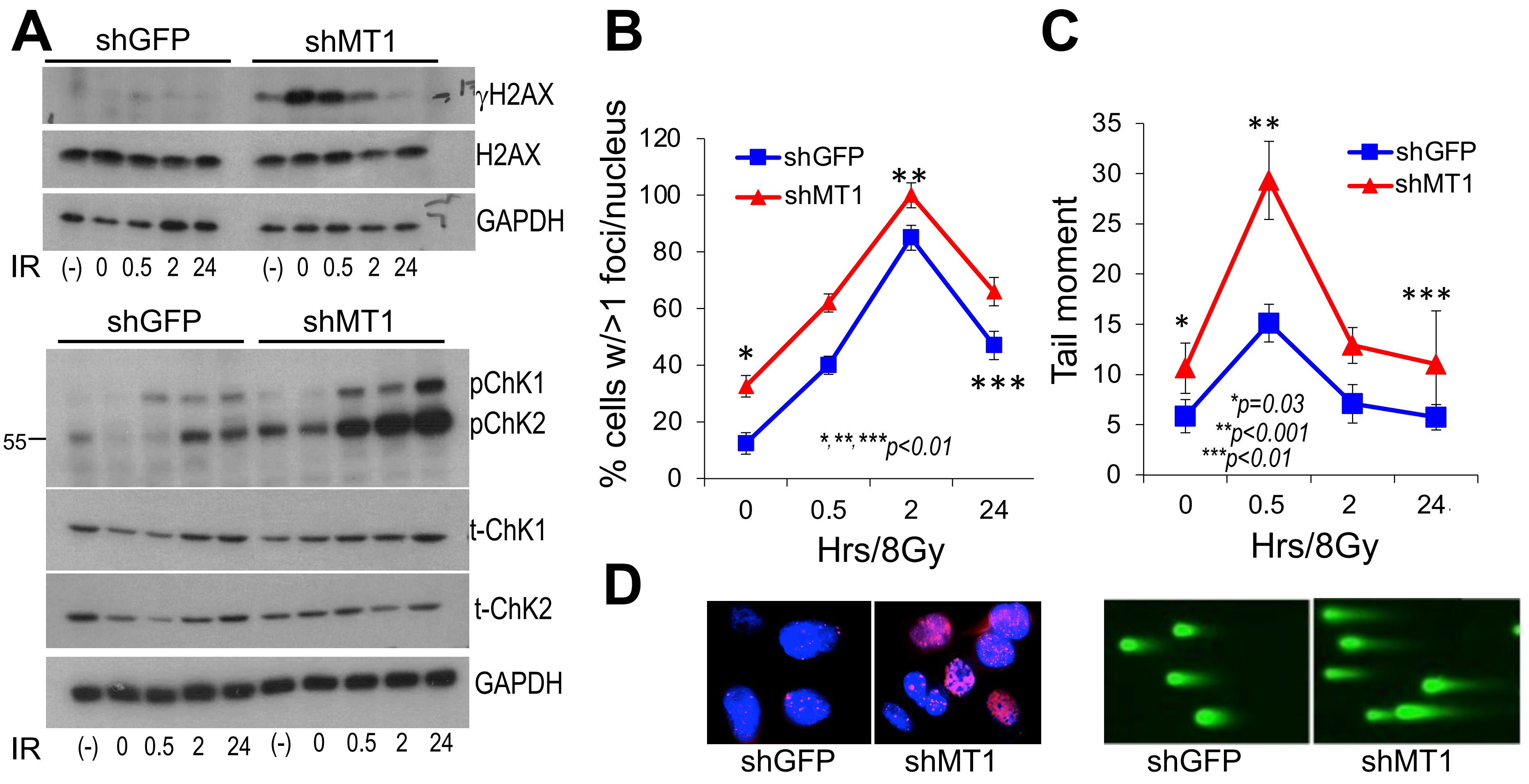

Suppl. Fig. 8: A) gH2AX, total H2AX, phosphorylated (active) and total ChK1 and 2 in 821-GSCs after 6Gy IR (one dose). Time course after radiation, delivered at T0, is shown). **B)** quantification of γH2AX foci in 821-GSCs cells expressing shGFP or shMT1-MMP. **C)** Tail moment of 821-GSCs cells expressing shGFP or shMT1-MMP. Data are the mean of two independent experiments. **D)** Representative pictures of γH2AX foci and comets in shGFP and shMT1-MMP expressing cells at time 0. At least 50 nuclei per field were counted, for a total of 10 fields per slide (n= 3 slides per condition).

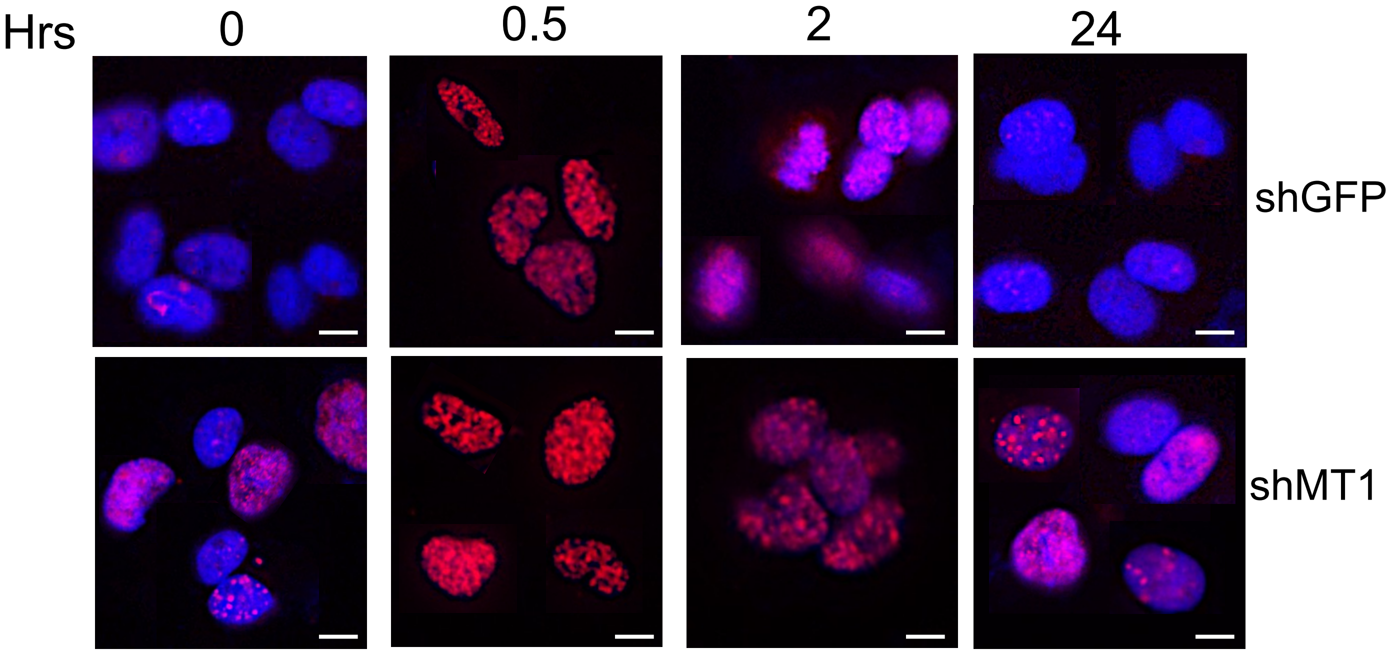

Suppl. Fig. 9: representative pictures of γH2AX foci in 913-GSCs cells expressing shGFP or shMT1-MMP after radiation.

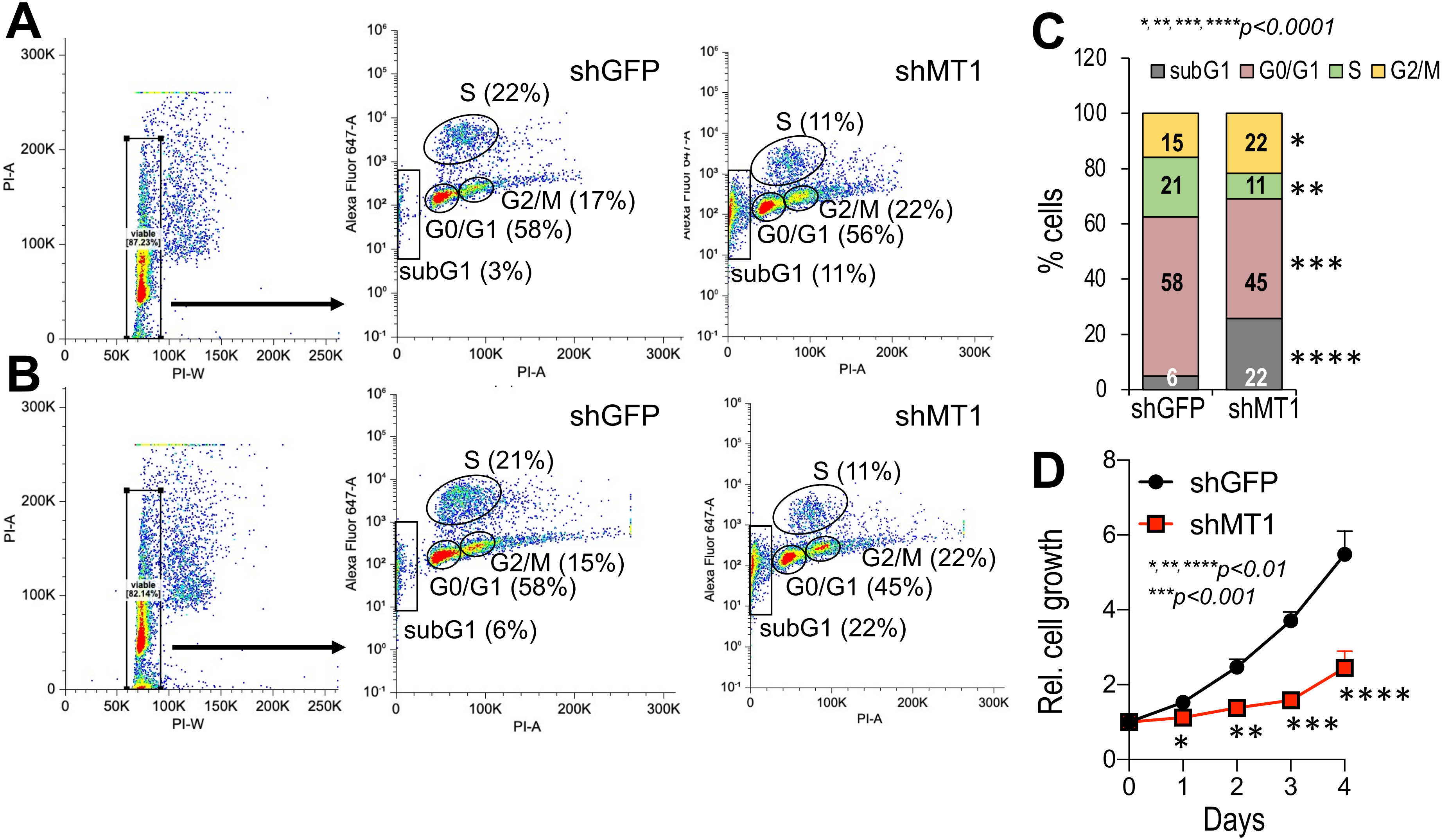

Suppl. Fig. 10: gating strategy for 913-GSCs (**A**) and 821-GSCs (**B**) showing representative cell cycle plots with the average cell percentage for each phase, in shGFP and shMT1-MMP expressing cells. **C)** % of 821-GSCs in the different phases of the cell cycle. **D)** relative cell growth of 821-GSCs expressing shGFP or shMT1. Time 0 normalized to 1. Means are the average of three independent experiments.

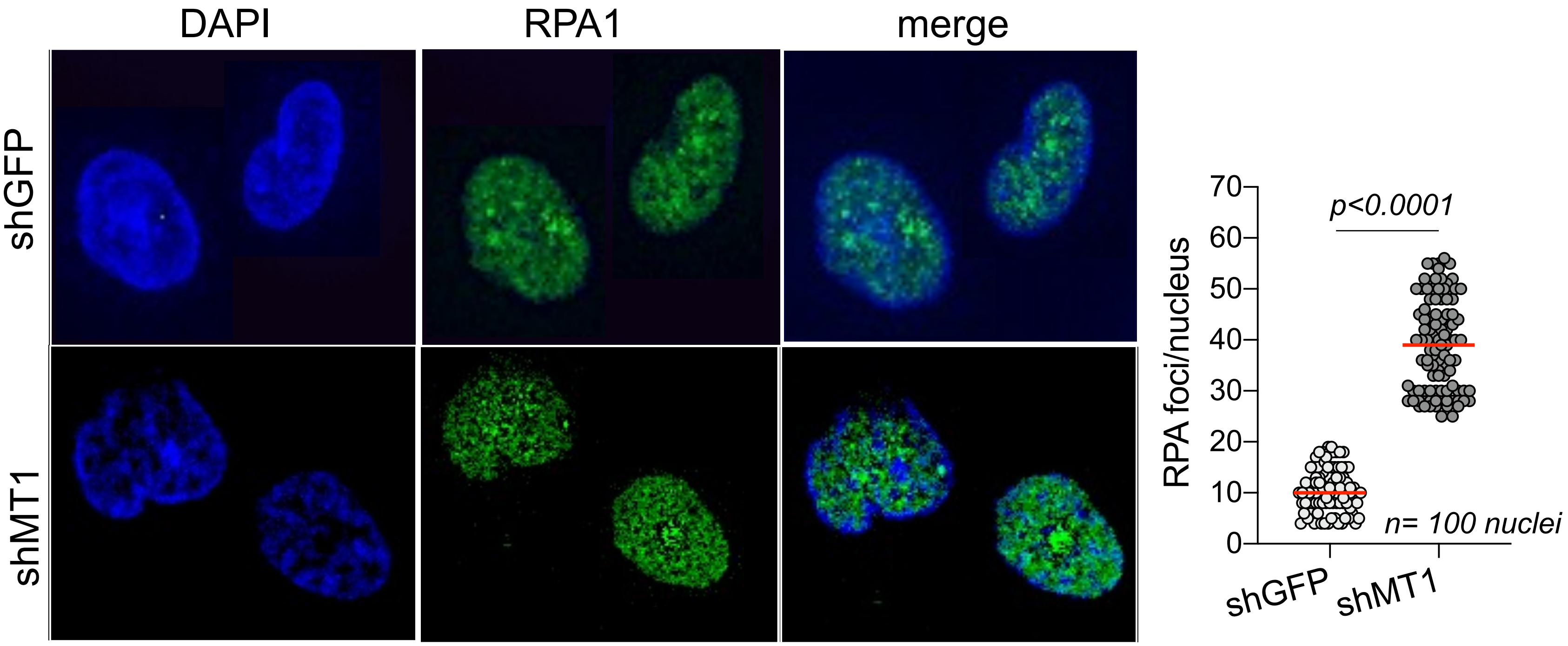

Suppl. Fig 11. RPA1 foci in shGFP and shMT1-MMP expressing 913-GSCs. DAPI was used as counterstain. Foci were counted in 100 nuclei per group in at least 5 slides. Values are the mean among two independent experiments.

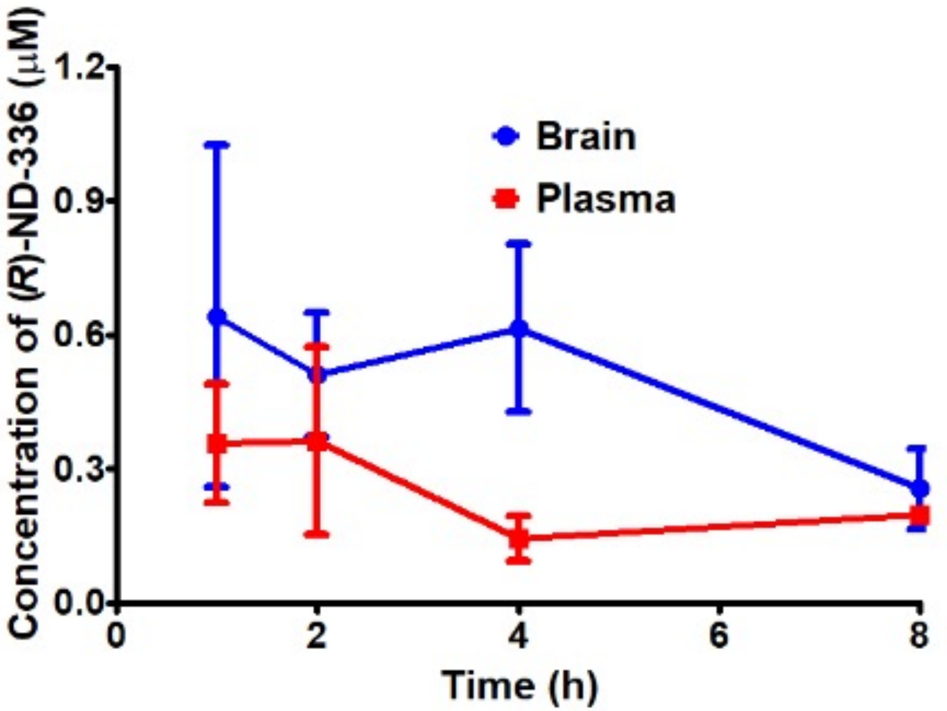

Suppl. Fig 12. Plasma (red) and brain (blue) concentrations after a single 10 mg/kg subcutaneous dose of ***(R)*-ND336** to mice (n=2 mice per time point). *(R)*-ND336 brain concentration is 255 nM at 8 hours.

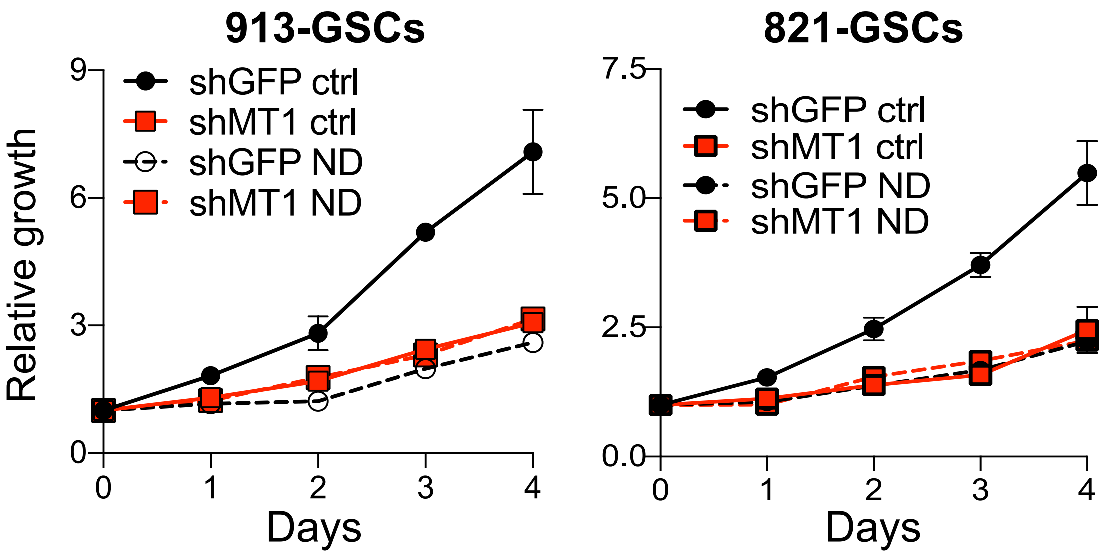

Suppl. Fig. 13: Relative cell growth of 913-GSC and 821-GSCs expressing shGFP or shMT1 and treated with DMSO (Ctrl) or 0.3uM (R)-ND336. Time 0 normalized to 1. Data are the average of two independent experiments. $shGFP_{ctrl}$ vs $shGFP_{ND}$, $p<0.001$; $shGFP_{ctrl}$ vs $shMT1_{ctrl}$, $p<0.001$; $shGFP_{ND}$ vs $shMT1_{ND}$, n.s.

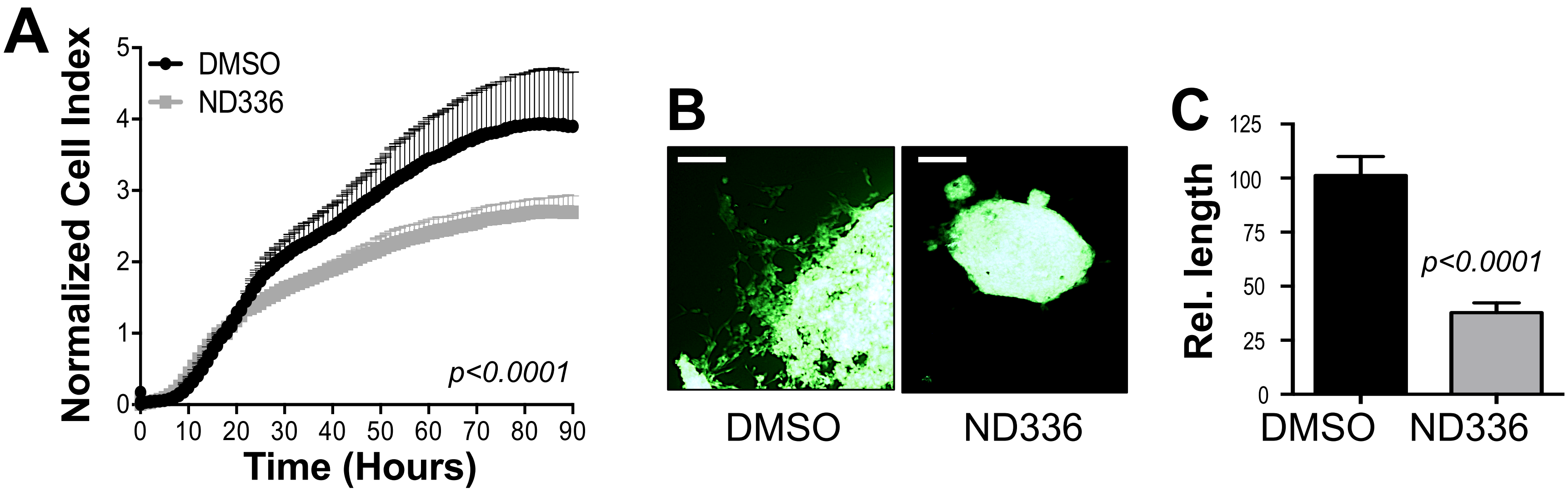

Suppl. Fig. 14: A) Invasion of 913-GSCs treated with 0.30 μM *(R)*-ND336 or vehicle (DMSO) Through a Matrigel/HA matrix (XCELLigence). **B)** Invasion of 913-GSCs treated with *(R)*-ND336 Through a 3D brain matrix. Scale bar 50um.**C)** quantification of invadopodia length of the cells in B. 100 spheroids per field were measured in 10 fields per slides (n=3 slides)

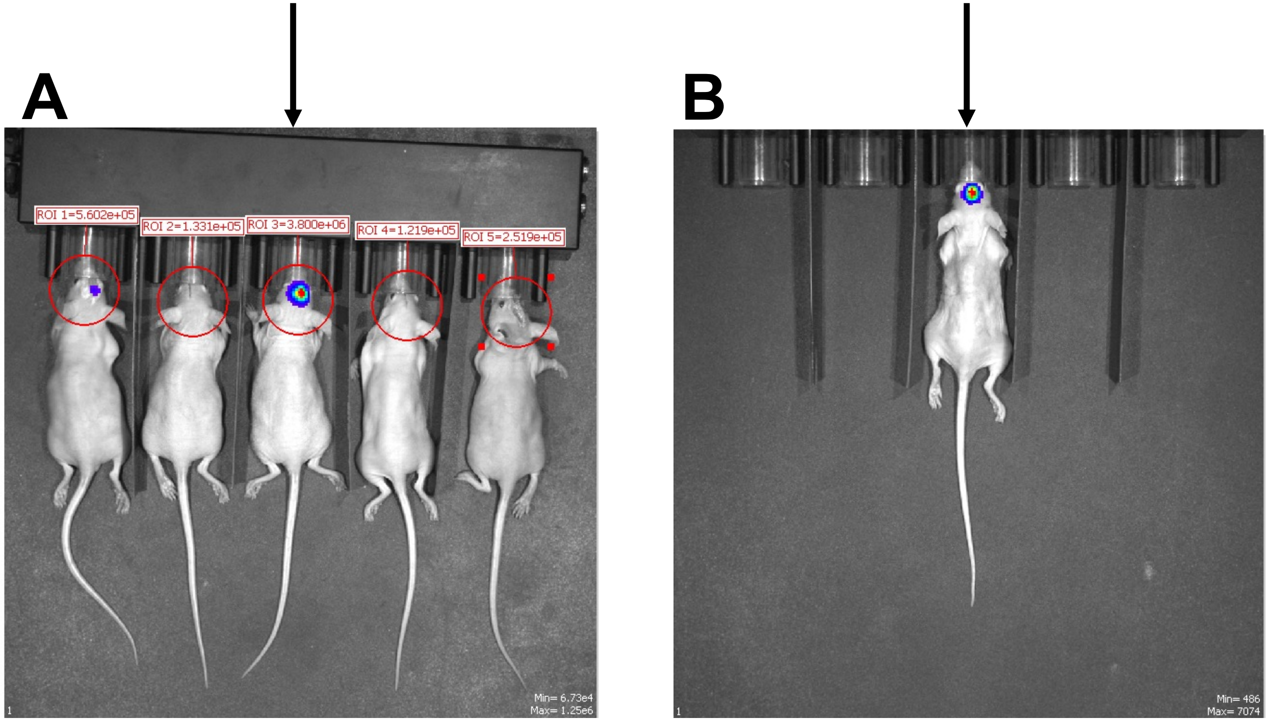

Suppl. Fig. 15: A) bioluminescence imaging (BLI) of nude mice bearing 913-GSCs one day post tumor cell inoculation in the brain. Arrow indicate last surviving animal (ROI at day1= $5.87x10^5$).**B)** BLI imaging of the last surviving mouse prior to sacrifice at day 365 (ROI at day 365= $7.4x10^8$). All BLIs were taken maintaining mice in the same order in the IVIS chamber.

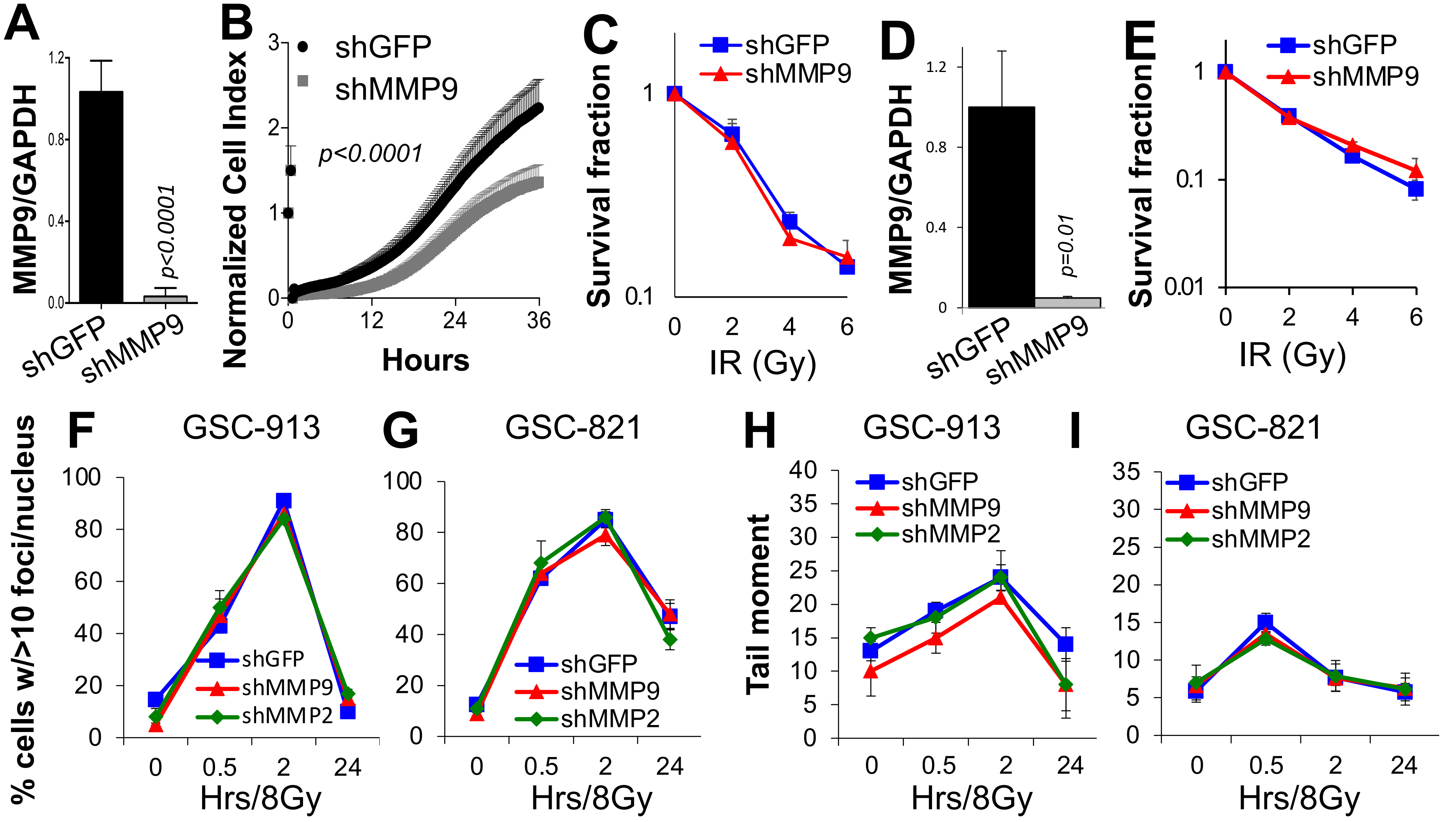

Suppl. Fig. 16: A) RT-PCR of 913-GSCs cells expressing shGFP or shMMP9. **B)** Invasion of the cells in A through a Matrigel/HA matrix (XCELLigence). **C)** Clnogenic assay of the cells in A subjected to increasing radiation doses. **D)** RT-PCR of 821-GSCs cells expressing shGFP or shMMP9. **E)** Clnogenic assay of the cells in D subjected to increasing radiation doses. **F)** γH2AX foci counted in the nuclei of 913-GSCs expressing shGFP or shMMP2 or shMMP9. **G)** tail moment of 913-GSCs expressing shGFP or shMMP2 or shMMP9. **H, I)** γH2AX foci and tail moment of 821-GSCs expressing shGFP, shMMP2 or shMMP9. At least 50 nuclei per field were counted, for a total of 10 fields per slide (n=3 slides per condition). Tail moment was determined in ≥100 comets/group.
